# Seedling microbiota engineering using bacterial synthetic community inoculation on seeds

**DOI:** 10.1093/femsec/fiae027

**Published:** 2024-03-19

**Authors:** Gontran Arnault, Coralie Marais, Anne Préveaux, Martial Briand, Anne-Sophie Poisson, Alain Sarniguet, Matthieu Barret, Marie Simonin

**Affiliations:** IRHS-UMR1345, Université d’Angers, INRAE, Institut Agro, SFR 4207 QuaSaV, 49071, Beaucouzé, France; IRHS-UMR1345, Université d’Angers, INRAE, Institut Agro, SFR 4207 QuaSaV, 49071, Beaucouzé, France; IRHS-UMR1345, Université d’Angers, INRAE, Institut Agro, SFR 4207 QuaSaV, 49071, Beaucouzé, France; IRHS-UMR1345, Université d’Angers, INRAE, Institut Agro, SFR 4207 QuaSaV, 49071, Beaucouzé, France; Groupe d’Étude et de Contrôle des Variétés et des Semences (GEVES), 49070, Beaucouzé, France; IRHS-UMR1345, Université d’Angers, INRAE, Institut Agro, SFR 4207 QuaSaV, 49071, Beaucouzé, France; IRHS-UMR1345, Université d’Angers, INRAE, Institut Agro, SFR 4207 QuaSaV, 49071, Beaucouzé, France; IRHS-UMR1345, Université d’Angers, INRAE, Institut Agro, SFR 4207 QuaSaV, 49071, Beaucouzé, France

**Keywords:** seed microbiota, seedling microbiota, transmission, microbiota engineering, Synthetic Community

## Abstract

Synthetic Communities (SynComs) are being developed and tested to manipulate plant microbiota and improve plant health. To date, only few studies proposed the use of SynCom on seed despite its potential for plant microbiota engineering. We developed and presented a simple and effective seedling microbiota engineering method using SynCom inoculation on seeds. The method was successful using a wide diversity of SynCom compositions and bacterial strains that are representative of the common bean seed microbiota. First, this method enables the modulation of seed microbiota composition and community size. Then, SynComs strongly outcompeted native seed and potting soil microbiota and contributed on average to 80% of the seedling microbiota. We showed that strain abundance on seed was a main driver of an effective seedling microbiota colonization. Also, selection was partly involved in seed and seedling colonization capacities since strains affiliated to Enterobacteriaceae and Erwiniaceae were good colonizers while Bacillaceae and Microbacteriaceae were poor colonizers. Additionally, the engineered seed microbiota modified the recruitment and assembly of seedling and rhizosphere microbiota through priority effects. This study shows that SynCom inoculation on seeds represents a promising approach to study plant microbiota assembly and its consequence on plant fitness.

## Teaser

Inoculation of common bean seed with diversified bacterial synthetic communities can induce deep modifications of both seed and seedling microbiota, even in living potting soil.

## Introduction

Plants are associated with many microorganisms that can modulate their fitness (Vannier et al. 2019, Arnault et al. 2023). In this context, microbiota engineering is gaining attention as a potential way to improve plant disease management (Li et al. 2021, Malacrino et al. 2022), plant resilience (Schmitz et al. 2022), and plant biomass (Liu et al. 2023) in a more sustainable agricultural framework (Trivedi et al. 2021). One way to modulate the composition of the plant microbiota is to assemble several cultured microorganisms (i.e. bacterial or fungal strains) in Synthetic Communities (SynComs). SynComs represent means to establish causality between microbiota composition and plant fitness (Vorholt et al. 2017) and could be designed to improve plant health (Shayanthan et al. 2022). To date, SynComs have been primarily applied on soil (Liu et al. 2021, Baas et al. 2016), rhizosphere (Li et al. 2021, Marín et al. 2021), root (Durán et al. 2018, Vannier et al. 2023), and phyllosphere (Chen et al. 2020).

Use of SynCom for microbiota engineering in agriculture is still facing some concerns including (i) stability of the SynCom over time, (ii) capacity of SynCom members to colonize plant tissues, and (iii) ability to compete with native communities (Rocca et al. 2021). This latter phenomenon is called community coalescence (Rillig et al. 2015, Rocca et al. 2021). It is generally difficult to predict the outcome of a coalescence event since the resulting community depends on both neutral (e.g. dispersal and drift) and niche-based processes (e.g. host selection and biotic interactions). One important parameter that could influence the outcome of community coalescence is the flow of individuals created by differences in community size, i.e. mass effect (Shmida and Wilson 1985). For instance, Chen et al. (2020) showed that SynCom concentration was a key factor that influenced both the SynCom stability in plant tissues and the microbial interactions within the SynCom. Hence, SynCom inoculation represents a promising tool in agriculture but more research is needed to improve their efficiency and yield more predictable coalescence outcomes with native communities.

Plant microbiota engineering using SynCom inoculation on seed is gaining attention as a potential way to reduce the amount of inoculum needed (Rocha et al. 2019). Indeed, through priority effect (i.e. the effect of order and timing of species immigration during community assembly) seed could be a relevant vector to transmit beneficial microbiota to seedlings and change the trajectory of plant microbiota assembly (Debray et al. 2022). Still, to date, only few studies have used SynComs on seed (Figueiredo dos Santos et al. 2021, Armanhi et al. 2021, Kaur et al. 2022, Simonin et al. 2023), despite the potential use of seed coating to deliver beneficial microorganisms to crops (Rocha et al. 2019). The first studies involving simple consortia (often composed of two strains) inoculated onto seeds have demonstrated the potential of this approach in enhancing seed germination and seedling phenotype (Srinivasan and Mathivanan 2009, Cassán et al. 2009). However, these consortia were composed of strains not derived from the seeds and the ecological processes associated with the assembly of the seed and seedling microbiota were not investigated. Additionally, seed microbiota has been shown to promote seedling growth and protection against fungal disease (Pal et al. 2022). Thus, seed-borne taxa represent an untapped microbial resource to improve plant protection and yield. Seeds harbor a specific microbiota with reduced microbial community size (i.e. number of individuals) and species richness (i.e. number of species) compared to other plant compartments (Guo et al. 2021) and show significant variations between seeds (Chesneau et al. 2022). Moreover, several studies report that only a fraction of the seed microbiota is transmitted to the seedlings (Rochefort et al. 2021, Walsh et al. 2021, Abdelfattah et al. 2023, Chesneau et al. 2022). In this sense, Walsh et al. (2021) argue that seed inoculants may exhibit reduced effectiveness in highly diversified and populated soil due to mass effect. On the contrary, Moroenyane et al. (2021) contend that through priority effect, the seed microbiota could be a promising candidate for microbiota engineering. Thus, the ecological processes involved in the assembly of the seedling microbiota need to be further investigated. In this study, we employed a SynCom approach on seeds to gain a better understanding of the role of these ecological processes, with a focus on mass effect and selection processes.

**Figure 1.**
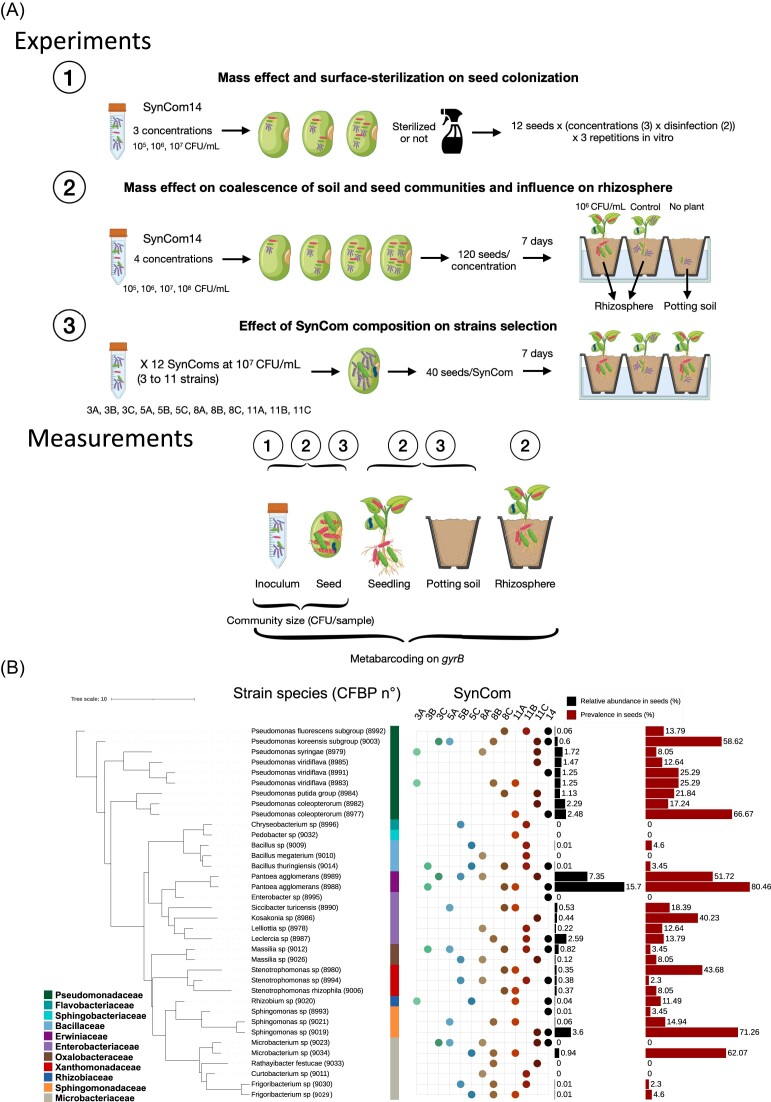
Design of the different experiments, strain selection, and SynCom compositions. (A) Overview of the different experiments. Experiment 1 was designed to test the influence of SynCom14 mass effect (composed of 14 bacterial strains) on surface-sterilized and unsterilized seeds using different concentrations (hypothesis 2). Experiment 2 was designed to study the influence of SynCom14 mass effect on seed and seedling microbiota assembly using different concentrations in a coalescence context with potting soil (hypotheses 2 and 4). Experiment 3 was set to study the influence of the inoculation of 12 different SynComs (with 3, 5, 8, or 11 bacterial strains) on seed and seedling microbiota assembly (hypothesis 3). (B) Phylogenetic tree of the 36 strains selected and composition of the 13 SynComs. SynCom14 was studied in experiments 1 and 2 and the others in experiment 3. The number in SynCom names indicates the SynCom richness. Relative abundance and prevalence of each strain in the original seed samples are plotted on the right side. Seven strains were selected while they were not detected using the metabarcoding approach. Panel A was created with Biorender.com.

We hypothesize that both seed and seedling microbiota can be drived by SynCom inoculation on seed (H1). To validate this hypothesis, we compared the inoculation impact of 13 bacterial SynComs on common bean seeds (*Phaseolus vulgaris L*.) (Fig. 1A) on seed and seedling microbiota assembly. We then tested the mass effect hypothesis proposing that SynCom colonization of seed and seedling increases with SynCom inoculum concentration (H2). Also, we hypothesize that through selection processes SynCom strains exhibit varying colonization capacities for both seeds and seedlings (H3). To examine this hypothesis, 12 SynComs of variable species richness and composition were designed and seed inoculated (Fig. 1B). Finally, we hypothesized the priority effects of seed inoculum influencing the recruited communities of both seedling and rhizosphere (H4). To explore this hypothesis, we compared the effect of one SynCom on rhizosphere assembly and the effect of different SynComs on the recruited seedling communities.

## Materials and methods

### Plant material and constitution of the culture-based collection of seed bacterial strains

Eight genotypes (Flavert, Linex, Facila, Contender, Vanilla, Deezer, Vezer, and Caprice) of common bean (*P. vulgaris*) were grown on the field in 2020 by the National Federation of Seed Multiplicatiers (FNAMS) on two locations [Condom, Gers (43.956991, 0.392127) and Brain sur l'Authion, Maine-et-Loire (47.470532, −0.394526), France]. These seed samples were used to obtain a collection of bacterial isolates from both seeds and seedlings. To isolate strains from seedlings germinated under gnotobiotic conditions, 30 seeds per condition were grown in cotton soaked with 4 ml of sterile water (growth for 7 days at 25°C, 16 h of day, 8 h of night) and the seedlings were then crushed and homogenized with 2 ml of sterile water. The seeds were soaked in 2 ml of sterilized water per gram of fresh material at 4°C with agitation (220 r/m) overnight (∼16 h). Suspensions were plated on tryptic soy agar (TSA) 10% strength and incubated at 18°C for 7 days. Isolated colonies were then picked based on morphotypes and grown in TSB in 96-well plates for 4 days at 18°C. A total of 1276 strains from these different seed and seedling samples were collected and stored at −80°C in 40% glycerol. Genotyping of the 1276 isolates was performed by metabarcoding of the *gyrB* bacterial marker gene (Illumina MiSeq). To assess the representativeness of our strain collection, the total bacterial community composition of the seed (8 genotypes × 2 production sites × 3 replicates = 48 seed samples) and seedling (8 genotypes × 2 prod × 1 rep = 16 seedling samples) samples was characterized in parallel by metabarcoding of the *gyrB* marker following the protocol established by Barret et al. (2015) (see detailed methods below).

From the 1276 isolates, 36 strains were selected (and deposited in the CIRM-CFBP strain collection). These strains were selected according to two criteria: (i) abundance and prevalence of their *gyrB* sequences in the seed/seedling microbiota (Figure S1B, Supporting Information; Fig. 1B) and (ii) phylogenetic diversity. For the latter criterion, maximum-likelihood phylogenetic inference was based on the alignment of 1276 *gyrB* sequences (Figure S1A, Supporting Information). The selected strains represented 46.2% of the relative abundance of the seed microbiota and 11 distinct phylogenetic families (Fig. 1B). The DNA of these bacteria was extracted using the Wizard® Genomic DNA Purification Kit (Promega). Genomes were sequenced at the BGI (China) using DNBSEQ technology and assembled with Spades v3.15.3 using the default k-mer parameters (-k 21, 33, 55, 77, 99, and 127) and the following options: –cov-cutoff auto, –isolate (Prjibelski et al. 2020). Genomes are available on NCBI using the BioProject ID PRJNA1041598.

### Design and inoculation of SynCom on seeds

All experiments were performed using a commercial seed lot of Flavert genotype from Vilmorin-Mikado (France). Strain population sizes and SynCom sizes (measured in colony-forming units—CFU) of the inocula and inoculated seeds were verified by dilution and plating on 10% strength TSA. Throughout the manuscript, we have chosen to consistently use the term community size when referring to the number of individuals (i.e. the number of bacterial cells estimated using CFU). For each experiment, the control condition corresponded to the inoculation with sterile water. Control seeds were characterized using three batches of 25 seeds to obtain sufficient DNA for extraction.

Mass effect and surface-sterilization on seed colonization (Experiment 1)

The first experiment was designed to adjust the SynCom inoculation protocol for bean seeds (experiment 1, Fig. 1A). In particular, we tested the influence of mass effects (using variable inoculum concentration) and seed surface disinfection on SynCom’s capacity to colonize seeds using a SynCom composed of 14 strains (hereafter SynCom14). SynCom14 was designed to be as representative of the common bean seed microbiota as possible. The majority of bacterial families (9 out of 11) of the selected strains were included and SynCom14 ASVs accounted for a cumulative relative abundance of 17.45% within the native common bean seed microbiota.

Seeds were surface-sterilized using this protocol: sonicated for 1 min (40 Hz), soaked during 1 min in 96° ethanol, 5 min in 2.6% sodium hypochlorite, and 30 s in 96° ethanol, and rinsed three times with sterile water. SynCom14 was inoculated at different concentrations: 10^5^, 10^6^, and 10^7^ CFU/ml. To do so, each strain was resuspended in water by scratching a 48 h culture of 10% TSA. Each strain was then adjusted to an OD (600 nm) of ∼0.1 and SynCom was prepared by adding equivolume of each strain. Serial dilutions were made to obtain the final concentrations and strain and SynCom concentrations were checked using dilution and plating on 10% strength TSA. SynCom inoculations were performed by placing the seeds in a sterile container and adding 2 ml of inoculum (sterile water for control) per gram of seed for 30 min under agitation (70 r/m) at 18°C. Excess inocula were then removed using a sterile strainer, and seeds were dried for 30 min under laminar flow. The microbial community size that binds the seed post-inoculation (CFU/seed) was measured using 36 seeds per condition (12 seeds per replicate × 3 independent experiment replicates). Inoculated seeds were individually soaked in 2 ml of sterile water at 4°C under agitation (220 r/m) overnight (∼16 h). Suspensions were then plated on TSA 10% strength and incubated at 18°C for 4 days before counting CFUs (Fig. 1A). Microbiota profiling was performed on 24 seeds per condition (8 seeds × 3 biological replicates).

Mass effect on coalescence of soil and seed communities and influence on rhizosphere microbiota (Experiment 2)

A second experiment aimed to study mass effects between seed and potting soil communities during the establishment of seedling microbiota in a coalescence framework (experiment 2, Fig. 1A). To do so, nonsterile potting soil (∼ 10^7^ CFU/g) was used and SynCom14 was inoculated as explained before at four different inoculum concentrations (10^5^, 10^6^, 10^7^, and 10^8^ CFU/ml). A total of 120 seeds per condition were germinated in nonsterile potting soil during 7 days in a growth chamber (16 h day at 23°C, 8 h night at 20°C, 70% humidity) to assess SynCom14 contribution to seedling microbiota. The microbial community size that binds the seed post-inoculation (CFU/seed) was measured as described before for 8 seeds per condition. We also assessed the effects of SynCom inoculation on seeds on the rhizosphere microbiota. To do so, the potting soil bacterial communities were studied without seedling (no seedling), with 7 day seedlings that have not been inoculated (control seedling), and with seedlings inoculated with SynCom14 at 10^6^ CFU/ml. The adhering soil to seedling roots was considered as rhizosphere and conserved at −80°C before DNA extraction. Microbiota profiling was done on 8 seeds, 16 seedlings and 4 potting soil/rhizosphere per condition.

Effect of SynCom composition on strain selection (Experiment 3)

A third experiment was designed to test the effect of niche-based selection using several SynCom compositions and of four levels of richness (3, 5, 8, and 11 strains) to match the natural bacterial diversity observed on common bean mature seeds (Chesneau et al. 2022). For each given richness, strains were drawn randomly and without replacement using a pool of 33 strains [see Fig. 1C for SynComs composition and Figure S1 (Supporting Information) for strain selection]. The 33 strains were chosen to avoid including two strains with the same ASV, ensuring distinct *gyrB* ASV for strain tracking.

A total of 40 seeds per condition were inoculated using a 10^7^ CFU/ml suspension following the same procedure described before and let grown in the same condition as described for experiment 2. Microbiota profiling was done on the inocula, 8 seeds and 8 seedlings per condition. We also measured the microbial community size that binds the seed post-inoculation (CFU/seed) as described before for 8 seeds per condition. Even if SynComs were inoculated at 10^7^ CFU/ml, the seeds post-inoculation showed different community sizes (Kruskal–Wallis, *P*-value < .001; Figure S9A, Supporting Information). This outcome was used to validate the hypothesis of mass effects (Fig. 3D), as previously demonstrated using experiment 2 (Fig. 3A).

### Plant growth, DNA extraction, and *gyrB* gene sequencing taxonomic classification

The following metabarcoding approach was performed on the inocula, inoculated seeds and seedlings of the different SynComs. For inocula, 200 µl of each fresh inoculum was instantly stored at −80°C before DNA extraction. For inoculated seeds, individual seeds were soaked in 2 ml of water overnight (∼16 h) at 6°C under agitation (220 r/m), 200 µl of each suspension obtained was stored at −80°C before DNA extraction and suspension were plated to assess community size on seeds.

After 7 days of growth (BBCH stage 12, two full leaves unfolded), seedling roots were cleaned of potting soil excess by hand shaking and using sterilized water. Whole seedling samples were first crushed with a roller. Then 2 ml of sterilized water was added and the samples were ground for 30 s using a stomacher. DNA was extracted using 200 µl of the crushed suspension with the NucleoSpin® 96 Food kit (Macherey-Nagel, Düren, Germany) following the manufacturer’s instructions. For potting soil and rhizosphere characterization, four replicates of 200 mg per condition were extracted using DNA PowerSoil kit from Qiagen following the manufacturer’s instructions.

To ensure accurate strain traceability, we conducted a comparison between *gyrB* and 16S (V4 region) utilizing genomic data [see Figure S8 (Supporting Information) legend for more details]. Notably, while 19 strains exhibited identical 16S (V4) sequences, only two strains shared the same *gyrB* sequence. Consequently, we opted for the utilization of *gyrB* to effectively track our strains across inocula, seeds, seedlings, and rhizosphere samples. The first PCR was performed with the primers gyrB_aF64/gyrB_aR553 (Barret et al. 2015), which target a portion of *gyrB* gene in bacteria. PCR reactions were performed with a high-fidelity Taq DNA polymerase (AccuPrimeTM Taq DNA polymerase Polymerase System, Invitrogen, Carlsbad, California, USA) using 5 µl of 10X Buffer, 1 µl of forward and reverse primers (100 µM), 0.2 µl of Taq and 5 µl of DNA. PCR cycling conditions were done with an initial denaturation step at 94°C for 3 min, followed by 35 cycles of amplification at 94°C (30 s), 55°C (45 s) and 68°C (90 s), and a final elongation at 68°C for 10 min. Amplicons were purified with magnetic beads (Sera-MagTM, Merck, Kenilworth, New Jersey). The second PCR was conducted to incorporate Illumina adapters and barcodes. The PCR cycling conditions were: denaturation at 94°C (2 min), 12 cycles at 94°C (1 min), 55°C (1 min), and 68°C (1 min), and a final elongation at 68°C for 10 min. Amplicons were purified with magnetic beads and pooled. Concentration of the pool was measured with quantitative PCR (KAPA Library Quantification Kit, Roche, Basel, Switzerland). Amplicon libraries were mixed with 10% PhiX and sequenced with three MiSeq reagent kits v2 500 cycles (Illumina, San Diego, California, USA). A blank extraction kit control, a PCR-negative control and PCR-positive control (*Lactococcus piscium*, a fish pathogen that is not plant-associated) were included in each PCR plate. The raw amplicon sequencing data are available on the European Nucleotide Archive (ENA) with the accession number PRJEB59714.

The bioinformatic processing of the amplicons originating from the bacterial strain collection and SynCom experiments was performed in R. In brief, primer sequences were removed with cutadapt 2.7 (Martin 2011) and trimmed fastq files were processed with DADA2 v1.22.0 (Callahan et al. 2016). Chimeric sequences were identified and removed with the removeBimeraDenovo function of DADA2. Amplicon Sequence Variant (ASV) taxonomic affiliations were performed with a naive Bayesian classifier (Wang et al. 2007) with our in-house *gyrB* database (train_set_gyrB_v5.fa.gz) available upon request. Unassigned sequences at the phylum level and *parE* sequences (a *gyrB* paralog) were filtered. Then, to remove singleton coming from potential sequencing errors, only ASVs with a minimum of 20 reads and present in at least two different samples were retained for experiments 2 and 3 (three reads in at least three samples for experiment 1). These different filtering thresholds were set after multiple tests. Data filtering for experiments 2 and 3 was set to be more stringent after the observation of multiple unaffiliated sequences, which artificially increased the richness of the dataset. These ASVs were coming from the potting soil samples and might reveal some PCR error, maybe due to chemical interactions between the polymerase and persistent soil molecules. To track our SynCom strains, only ASVs with 100% of identity were considered to be our strains. Some single-nucleotide polymorphisms were identified for some strains and were artificially increasing inoculum richness. They were, thus removed before downstream analyses. All R scripts employed in this work are available on GitHub (https://github.com/GontranArnault/BeanSeedSynCom2023).

### Statistical analyses and microbiota analysis

Microbial community analyses were conducted using the Phyloseq package v1.44.0 (Mc Murdie and Holmes 2013) using R. Figures were visualized using ggplot2 v3.4.3. Alpha diversity analyses were performed with a coverage-based rarefaction as recommended by Chao and Jost (2012) using iNEXT package v2.6.4 (Hsieh et al. 2016). Beta diversity analyses were made using Bray–Curtis distance and permutational multivariate analysis of variance [adonis2 function of vegan v2.6.4 (Oksanen et al. 2019), 999 permutations] after a rarefaction at 10 000 reads per sample for experiments 1 and 3 and 6000 reads for experiments 2 (see rarefaction curves in Figure S2, Supporting Information). Beta diversity was visualized using Principal Coordinate Analysis (PCoA). For Fig. 6(A), ASVs of the SynCom strains were removed to visualize the recruited communities’ assembly. For mean comparisons, ANOVA followed by *post hoc* Tukey tests were conducted when the conditions of application were met. Otherwise, Kruskal–Wallis followed by pairwise Wilcoxon tests were conducted. *P*-values were corrected using Benjamini–Hochberg method when multiple comparisons were conducted.

To assess the relative contribution of native seed microbiota, potting soil and SynComs, a microbial source tracking analysis was conducted using the fast expectation–maximization microbial source tracking FEAST v0.1.0 package with a maximum iteration of 1000 (Shenhav et al. 2019). FEAST employed a microbial community as the sink and multiple potential sources to determine the respective contributions of each potential source to the sink microbial community. Control seed, inoculated seed and potting soil microbiota were considered as sources of microorganisms and seedling were considered as sink. The algorithm also identified an unexplained fraction, named ‘unknown’ source, which represented strains found in the sink but originating from potential unsampled sources.

The phylosignal package v1.3 (Keck et al. 2016) was used to confirm the observed phylogenetic pattern between strain families and their colonization capacity of both seed and seedling. To do so, a phylogenetic tree was constructed using automlst (commit b116031) with default parameters and 1000 bootstrap replicates (-bs 1000) (Alanjary et al. 2019). The local indicator of Phylogenetic Association index (lipaMoran) was calculated using the lipaMoran function. This index allowed us to test the positive or negative autocorrelations between the measured parameters (relative abundance on seed, seedling, and their ratio) and the phylogenetic position of a given strain. Fig. 1(B) was assembled using iTol.

Changes in the relative abundance of bacterial communities of rhizospheres from experiment 2 were evaluated employing a linear model through the CornCob package v0.3.2 using taxatree_models function (after a log2 transformation) and taxatree_plots function for visualization.

## Results

### Seed colonization by SynCom depends on both mass effect and SynCom composition but not seed disinfection

**Figure 2.**
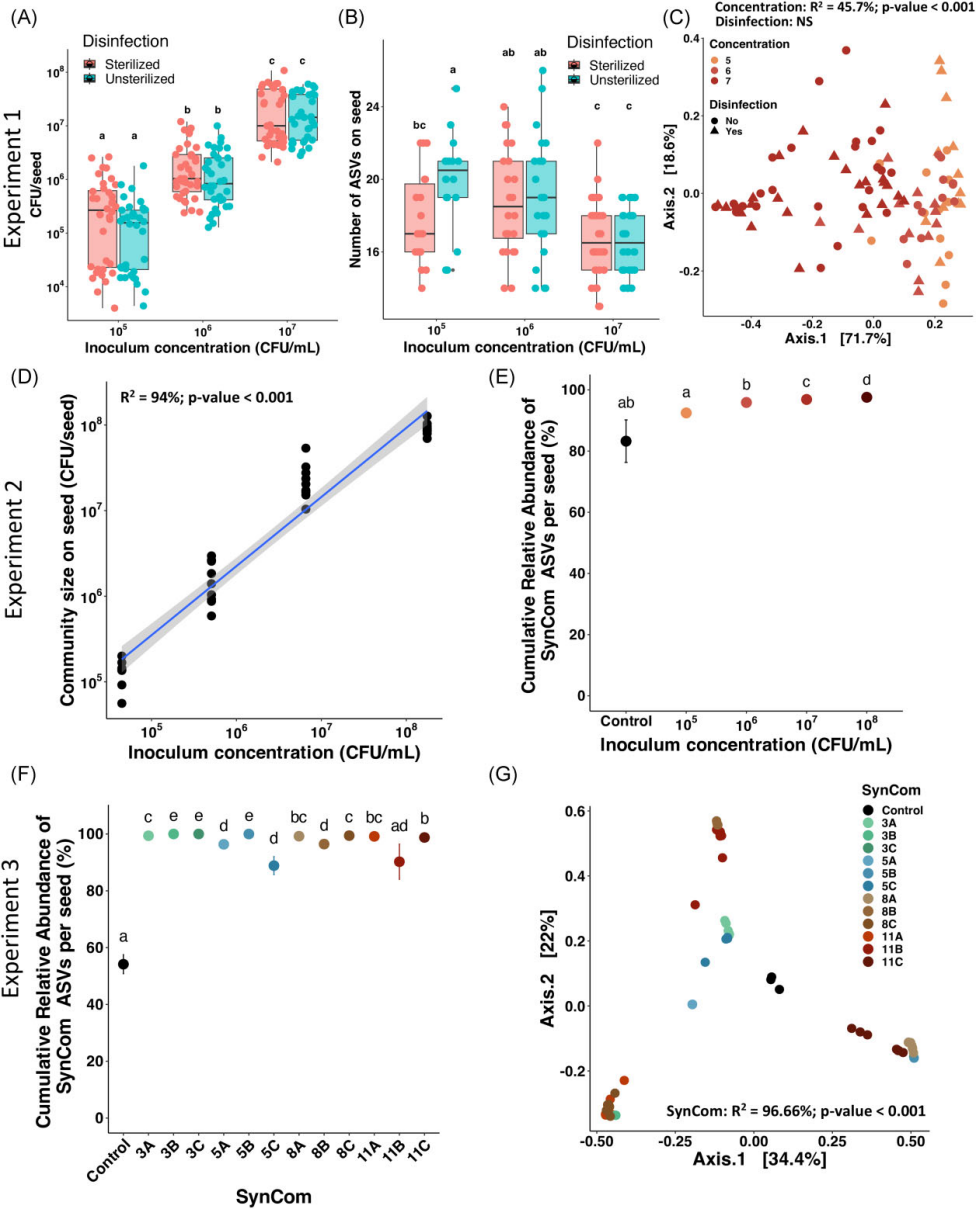
Effects of seed sterilization, SynCom mass effect and composition on seed microbiota assembly. Experiment 1: (A) community size on seed (CFU/seed) of SynCom14 in function of inoculum concentration (CFU/ml) and seed disinfection for the experiment 1 (36 seeds/treatment). The different letters represent the results of a *post hoc* Tukey HSD test. (B) Number of ASVs detected on seeds inoculated with SynCom14 in experiment 1 depending on inoculum concentration and seed disinfection (24 seeds/treatment). The different letters represent the results of a pairwise Wilcoxon test. (C) Influence of SynCom14 concentration and seed disinfection on seed bacterial community structure visualized through a PCoA ordination based on Bray–Curtis distances (PERMANOVA test; Disinfection effect: nonsignificant, Concentration: *R*^2^ = 45.7%, *P*-value < .001). Experiment 2: (D) linear model between community size on seed (CFU/seed) of SynCom14 in function of inoculum concentration (CFU/ml) for the experiment 2 (*R*^2^ = 94%, *P*-value < .001, 8 seeds/treatment). (E) Cumulative relative abundance of SynCom14 ASVs (SynCom colonization) in seeds from experiment 2 depending on inoculum concentration (8 seeds/treatment). The different letters represent the results of a pairwise Wilcoxon test. Experiment 3: (F) cumulative relative abundance of SynComs ASVs from experiment 3 (SynCom colonization) in seeds (8 seeds/treatment). The different letters represent the results of a pairwise Wilcoxon test. (G) Influence of SynCom composition of experiment 3 on seed bacterial community structure visualized through a PCoA ordination based on Bray–Curtis distances (PERMANOVA; SynCom condition: *R*^2^ = 96.66%, *P*-value < .001, 8 seeds/treatment).

A first SynCom, composed of 14 bacterial strains representative of the taxonomic diversity of our strain collection (SynCom14), was inoculated at three different concentrations on either surface sterilized or unsterilized bean seeds (Fig. 1, Exp1). Seed disinfection did not influence the number of CFU on seed after SynCom14 inoculation (Fig. 2A). All ASVs of SynCom14 were found on inoculated seeds. Furthermore, a few additional ASVs native to the seeds were detected (Fig. 2B). They accounted for less than 4% of the relative abundance and mainly belonged to Erwiniaceae, Pseudomonadaceae, and Enterobacteriaceae (Figure S10, Supporting Information). Bacterial richness (number of ASVs) was similar between native and disinfected seeds except for the lowest inoculated SynCom14 concentration (10^5^ CFU/ml) where disinfection reduced the number of ASVs (Wilcoxon test, *P*-value < .05, Fig. 2B). Finally, seed community composition was significantly impacted by SynCom concentrations (PERMANOVA, *R*^2^ = 45.7%, *P*-value < .001), while seed disinfection was not driving changes in community composition (Fig. 2C).

In a second independent experiment with nonsterilized seeds, changes in community sizes were correlated with the initial SynCom14 concentrations (Linear model, *R*^2^ = 94%, *P*-value < .001, Fig. 2D). SynCom strains’ ASVs were detected on control seed samples (83% of relative abundance), as they are members of the native bean seed microbiota (Figs 2E and 4). The ASVs corresponding to the strains assembled in the SynCom14 represented on average 96% of the cumulative abundance of bacterial taxa detected on seeds, ranging from 92% at the lowest concentration (10^5^ CFU/ml) to 98% at the highest concentration (10^8^ CFU/ml) (Fig. 2E).

In a third experiment, 12 SynComs were designed with four gradual levels of richness (3, 5, 8, and 11 strains). ASVs of the corresponding strains ranged from 89% (SynCom 5C) to 99.9% of the seed relative abundance (SynCom 3C, Fig. 2F). As expected, seed community composition was significantly explained by the SynCom inoculation (PERMANOVA, *R*^2^ = 96.7%, *P*-value < .001) (Fig. 2G).

Overall, these results showed that seed microbiota compositions were modified by the SynCom inoculation and that SynCom concentration and composition were the main drivers of the overall seed composition.

### Seedling colonization by SynComs is driven by mass effects and initial SynCom composition

**Figure 3.**
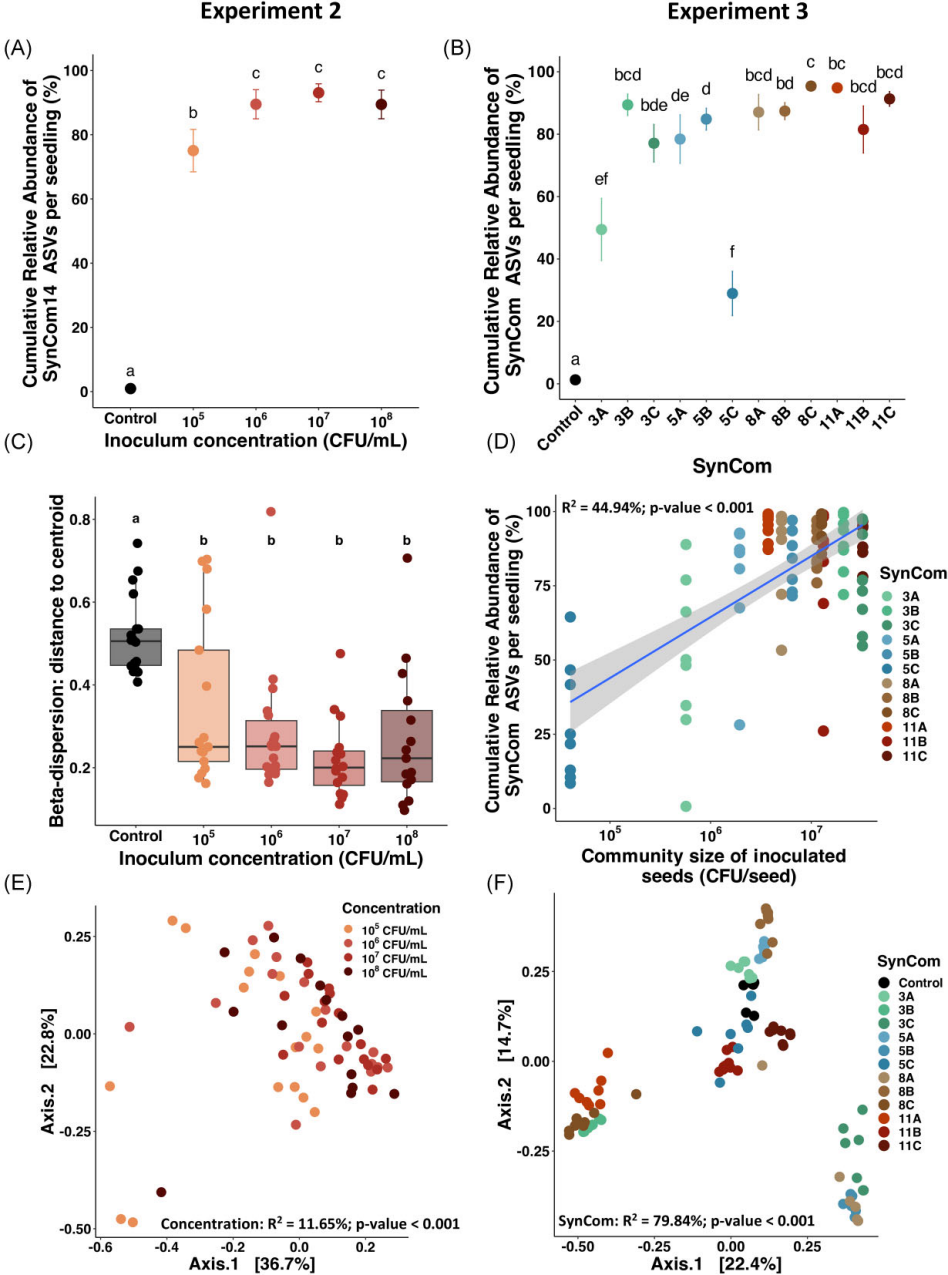
Influence of SynCom mass effect and composition on seedling microbiota assembly. (A) and (B) Cumulative relative abundance of SynComs ASVs (SynCom colonization) in seedlings from experiments 2 (A) (16 seedlings/treatment) and 3 (8 seedlings/treatment) (B). The different letters represent the results of a pairwise Wilcoxon test. (C) Beta-dispersion analysis (distance to centroid) of seedlings inoculated with SynCom14 (experiment 2). The different letters represent the results of a *post hoc* Tukey HSD test. (D) Linear model between the cumulative relative abundance of SynCom ASVs in seedlings and the mean community size of inoculated seeds from experiment 3 (*R*^2^ = 44.94, *P*-value < .001). (E) Influence of SynCom14 concentration on seedling bacterial community structure visualized through a PCoA ordination based on Bray–Curtis distances (PERMANOVA; concentration: *R*^2^ = 11.65%, *P*-value < .001). (F) Influence of SynCom composition from experiment 3 on seedling bacterial community structure visualized through a PCoA ordination based on Bray–Curtis distances. (PERMANOVA; concentration: *R*^2^ = 79.84%, *P*-value < .001).

To find out whether the compositional changes observed in the seed persist during emergence, seedling microbiota were characterized 7 days post SynCom inoculation. The level of seedling colonization was estimated by monitoring the cumulative relative abundance of ASVs corresponding to strains assembled in the SynComs. ASVs of SynCom14 were detected on control seedlings at a low level (< 1%). In inoculated condition, ASVs of SynCom14 inoculation represented on average 87% of the seedling relative abundance, ranging from 75% to 93% depending on inoculum concentration (Fig. 3A). A significant increase of SynCom14 ASVs’ relative abundance was observed between control, 10^5^ and 10^6^ CFU/ml before reaching a plateau (Wilcoxon test, *P*value < .05, Fig. 3A). Hence, seedling bacterial composition was successfully modified by SynCom inoculation. According to beta-dispersion (distance to centroid) the variability in seedling community structure was significantly reduced following SynCom14 inoculation compared to non-inoculated seeds (Fig. 3C). Moreover, seedling community composition was significantly influenced by SynCom14 inoculation (PERMANOVA; *R*^2^ from 35.9% at 10^5^ CFU/ml to 51.9% at 10^7^ CFU/ml, *P*-values < .001; Figure S6, Supporting Information) and concentration (PERMANOVA; *R*^2^ = 11.65%, *P*-value < .001, Fig. 3E).

To assess whether SynCom richness and composition could modify seedling microbiota composition, 12 SynComs of increasing strain richness were seed-inoculated at the same initial concentration (10^7^ CFU/ml; Figure S9A). On average, SynComs ASVs represented 80% of seedling microbiota (Fig. 3B). While SynComs colonizations of seedlings were more variable (29% for SynCom 5C to 95% for SynCom 8C), a very low influence of initial SynCom richness was detected (Linear model, R^2^ = 12.7%, *P*-value < .001; Figure S3, Supporting Information). In contrast the initial SynCom composition resulted in different seedling colonization (Pairwise Wilcoxon tests, *P*-value < .05, Fig. 3B). The most prominent example concerned the three SynComs composed of five strains with ∼80% of cumulative relative abundance for SynComs 5A and 5B and ∼30% for SynCom 5C (Fig. 3B). Even if SynComs were inoculated at 10^7^ CFU/ml, the seeds post-inoculation showed different community sizes (Kruskal–Wallis, *P*-value < .01; Figure S9, Supporting Information). Interestingly, the differences in seedling colonization were correlated with the community size of inoculated seeds (Linear model, *R*^2^ = 44.94%, *P*-value < .001, Fig. 3D). Thus, one important parameter that could predict bacterial colonization of seedling was its community size on seed. Seedlings inoculated with the same SynCom clustered together and the PERMANOVA confirmed that 79.84% (*P*-value < .001) of the variance was explained by the SynCom inoculation (Fig. 3D). Also, pairwise PERMANOVA confirmed that seedlings inoculated with the different SynComs showed distinct microbial communities, highlighting the efficiency of the method to manipulate seedling microbiota (Figure S7, Supporting Information).

Overall, these results showed that seedling microbiota of common bean could be deeply modified using SynCom inoculation on seeds and that this inoculation procedure greatly modified community composition observed on seedlings.

### Strains show heterogeneous transmission capacities from seed to seedling

**Figure 4.**
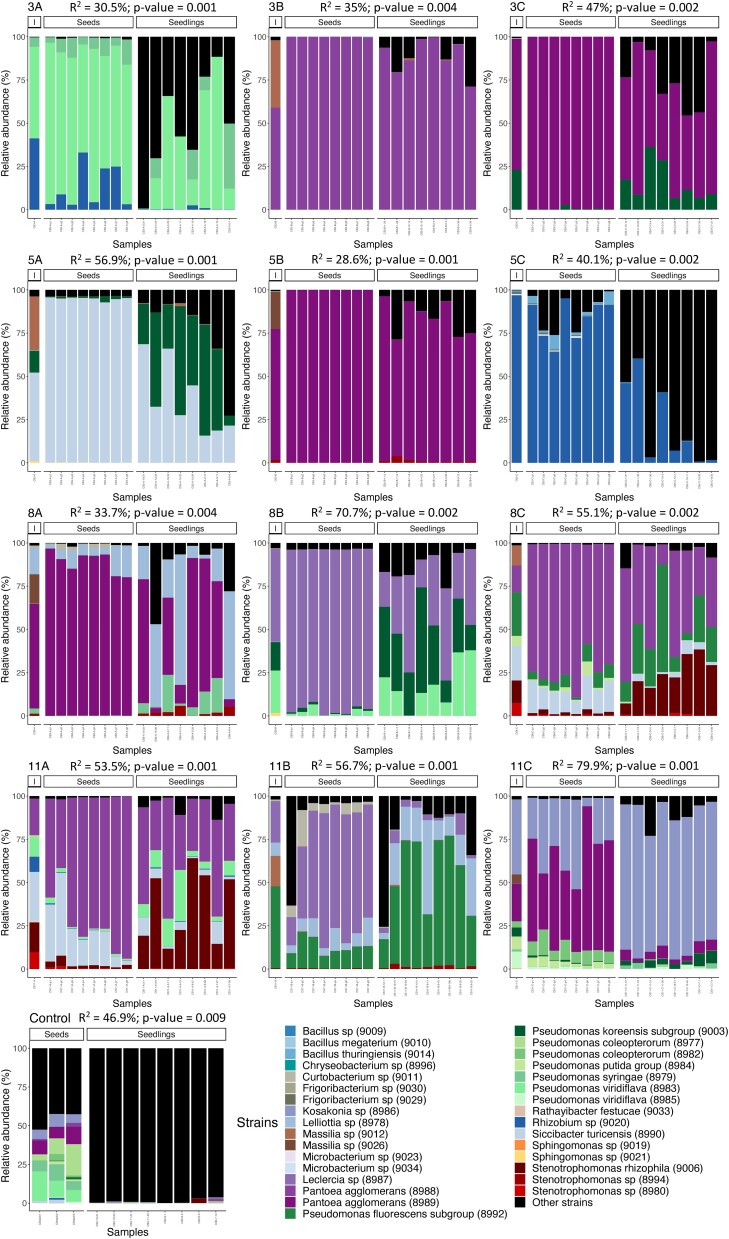
Influence of SynCom composition on taxonomic profiles of inocula, seeds, and seedlings from experiment 3. Relative abundance of inoculated strains in the inocula, seeds, and seedlings. Each stacked bar represents a sample. Only ASVs of the SynCom strains are colored, the black part represents uninoculated environmental taxa (e.g. potting soil and native seed microbiota). Per SynCom condition, one inoculum, 8 inoculated seeds, and 8 seedlings were characterized using amplicon sequencing of the *gyrB* gene. For the control condition (not inoculated), 3 seed batches of 25 seeds and 8 individual seedlings were characterized. For each treatment, a PERMANOVA was conducted to compare SynCom composition on seed *versus* seedling and reported in each corresponding panel.

Based on the unique *gyrB* ASV of each inoculated strain, we tracked the strain transmission from the inoculum to the seedling of experiment 3 (Fig. 4). All the strains’ ASVs were detected in at least two samples and were thus kept during the filtering process, except for the *Pedobacter sp* (CFBP9032) strain. Also, *Bacillus megaterium* (CFBP9010) was not detected in the inoculum of SynCom 8A and *Frigobacterium sp* (CFBP9029) was not detected in the inoculum of SynCom 8B but were further detected on seeds or seedlings (see Figure S9B, Supporting Information).

**Figure 5.**
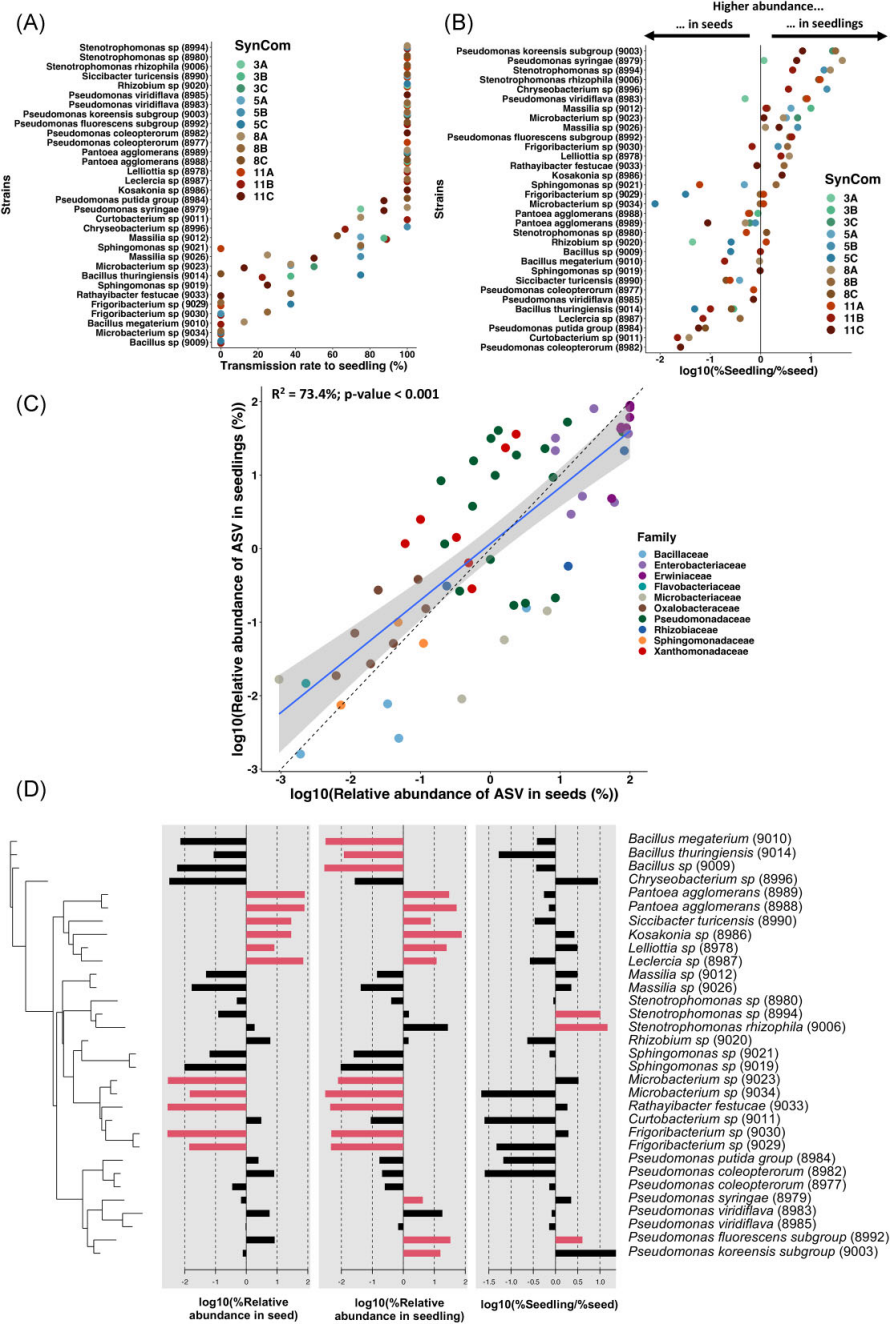
Transmission of each strain from seed to seedling. (A) Transmission rate to the seedlings of each strain in the different SynComs of experiment 3 (8 seeds/treatment). (B) Strain’s ability to colonize seedlings compared to their initial relative abundance on seeds, depending on the SynCom composition of experiment 3 (8 seeds and 8 seedlings/treatment). The following ratio was calculated to assess this trait: log10(% relative abundance on seedling/% relative abundance on seed). (C) Linear model between each ASV relative abundance on seeds compared to their relative abundance on seedlings (*R*^2^ = 73.4%, *P*-value < .001). y = x dashed line was plotted to see if an ASVs is enriched or depleted in seedlings compared to its relative abundance on seeds (above or under the y = x dashed line, respectively). (D) Phylogenetic pattern of strains colonization ability to colonize seeds and seedlings. Phylosignal package was used to test the significance of the observed phylogenetic signal. The red barplots show the significantly (*P*-value < .05) enriched or depleted strains based on local indicator of Phylogenetic Association index (lipaMoran), using the lipaMoran function.

The different SynCom panels confirmed the observation made on the overall community structures: seeds and seedlings inoculated with SynComs showed very different taxonomic profiles depending on the inoculated SynCom (Fig. 4). In particular, each SynCom condition showed very distinct taxonomic profiles even at strain level. Also, taxonomic compositions of the SynComs were significantly distinct between the seeds and the seedlings, highlighting the variability in strains’ ability to colonize the different habitats (PERMANOVA, *R*^2^ from 28.6% for SynCom 5B to 79.9% for SynCom 11C, Fig. 4). For instance, in SynComs 8C and 11A, *Stenotrophomonas rhizophila* (CFBP9006) had a reduced relative abundance from inoculum to seeds but then increased from seeds to seedlings. For a given strain in each SynCom, the transmission rate from seed to seedlings was assessed based on presence of ASV in each habitat (Fig. 5A). This transmission rate was not correlated with the relative abundance of the strain in the inoculum (Figure S9C, Supporting Information). We identified 16 strains that had a transmission rate to seedlings of 100% in each SynCom tested. These strains are from the genera *Kosakonia, Leclercia, Pantoea, Pseudomonas, Rhizobium, Siccibacter*, and *Stenotrophomonas*. On the other hand, *Microbacterium sp* (CFBP9034) and *Bacillus sp* (CFBP9009) strains had a transmission success of 0%. Some intermediate strains had interesting patterns: their transmission success was variable depending on the SynCom tested. For instance, *Sphingomonas sp* (CFBP9021) had a transmission rate of 75% in SynCom 8B and SynCom 5A and had a transmission rate of 0% in SynCom 11A. This example highlights the importance of strain interactions during seedling microbiota assembly (Fig. 5A).

To go further, we assessed the ability of each strain to colonize seedlings compared to their initial relative abundance on seeds, depending on the SynComs, using this ratio: log10(% Relative abundance on seedling/% relative abundance on seed) (Fig. 5B). Some strains were always found to be better seedling colonizers, including *Pseudomonas koreensis subgroup* (CFBP9003), *Pseudomonas syringae* (CFBP8979), *Stenotrophomonas sp* (CFBP8994), *S. rhizophila* (CFBP9006), *Chryseobacterium sp* (CFBP8996), *Massilia sp* (CFBP9012), and *Pseudomonas fluorescens subgroup* (CFBP8992). On the other hand, some strains were found to be better seed colonizers, including *Pseudomonas coleopterorum* (CFBP8982), *Curtobacterium sp* (CFBP9011), *Pseudomonas putida group* (CFBP8984), *Leclercia sp* (CFBP8987), *Bacillus thuringiensis* (CFBP9014), *Pseudomonas viridiflava* (CFBP8985), *P. coleopterorum* (CFBP8977), and *Siccibacter turicensis* (CFBP8990). Finally, some strains had variable behaviors depending on the SynCom composition. For instance, the *Rhizobium sp* (CFBP9020) was a better seed colonizer in the SynCom 3A and 5C but a better seedling colonizer in the SynCom 11A.

Additionally, we found that the seedling relative abundance of an ASV in a given SynCom was correlated with its relative abundance in seed (Linear model, *R*^2^ = 73.4%, *P*-value < .001, Fig. 5C). Thus, the seedling relative abundance of a given ASV could be predicted based on its relative abundance on seeds. This confirms at the strain level that high seed colonization leads to high seedling colonization. A phylogenetic pattern was observed in this correlation: Enterobacteriaceae and Erwiniaceae (purple) were highly abundant on both seed and seedling. Bacillaceae (blue) and Microbacteriaceae (grey) were depleted in seed and seedling microbiota (below the y = x dashed line), while *Stenotrophomonas* sp (CFBP8994) and *S. rhizophila* (CFBP9006) were enriched in seedling microbiota (red, Xandomonadaceae, above the y = x dashed line). This phylogenetic pattern was confirmed statistically using the local indicator of Phylogenetic Association index (*P*-value < .05, Fig. 5D). In particular, the three Bacillaceae exhibited a significantly low relative abundance in seedlings, whereas Erwiniaceae and Enterobacteriaceae strains were high colonizers of both seeds and seedlings (*P*-value < .05, Fig. 5D). Interestingly, Pseudomonadaceae were phylogenetically divided in two subgroups of differential colonization capacities: *P. syringae* (CFBP8979), *P. fluorescens subgroup* (CFBP8992), and *P. koreensis subgroup* (CFBP9003) were significantly more abundant in seedling while *P. putida group* (CFBP8984) and *P. coleopterorum* (CFBP8982 & CFBP8977) were less abundant in seedlings (Fig. 5D).

To conclude, we confirm that the relative abundance of a strain on seeds is a good predictor of its future relative abundance on seedling. However, strains showed different capacities of seed and seedling colonization depending on their phylogeny.

### SynCom inoculation influences seedling and rhizosphere microbiota recruitment from environmental sources

**Figure 6.**
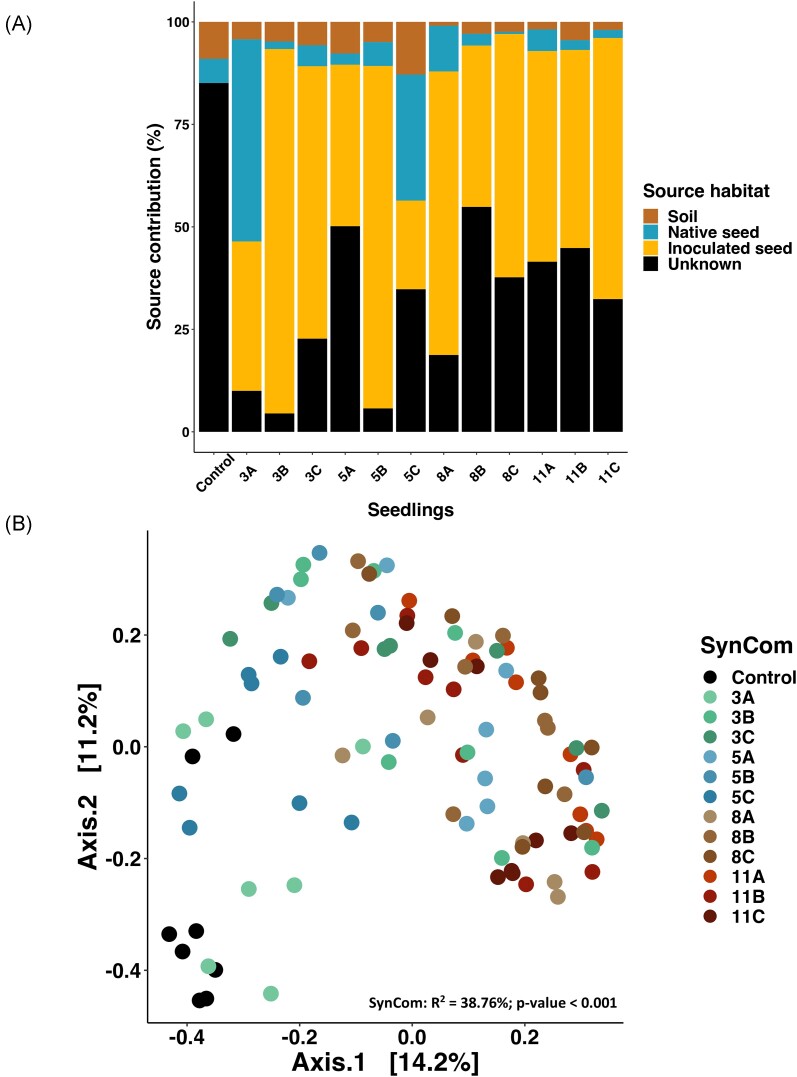
SynCom effect on environmental taxa recruitment of seedlings. (A) To assess the relative contribution of native seed microbiota, potting soil, and inoculated seed, a microbial source tracking analysis was conducted using FEAST. Control seed, inoculated seed, and potting soil microbiota were considered as sources of microorganisms and seedling were considered as sink. (See detailed boxplot for each source and SynCom and associated statistics in Figure S4, Supporting Information). (B) Influence of SynComs on seedling bacterial communities recruited from other sources (potting soil, air, and water) visualized through a PCoA ordination based on Bray–Curtis distances (PERMANOVA; SynCom: *R*^2^ = 38.76%, *P*-value < .001).

The fast expectation–maximization microbial source tracking algorithm (FEAST) was employed to evaluate the relative contributions of various inoculum sources to the seedling microbiota, including potting soil, native seed microbiota, and inoculated seed microbiota (Fig. 6A). Overall, the SynCom inoculation significantly decreased the unknown source proportion compared to control seedlings (Fig. 6A; Figure S4, Supporting Information). The native seed microbiota had a significantly increased contribution in SynComs 3A and 5C and a significantly decreased contribution in SynComs 3B, 5A, 8C, 11B, and 11C compared to the control (pairwise Wilcoxon, *P*-value < .05, Fig. 6A; Figure S4, Supporting Information). The potting soil contribution was on average 4.9% (ranging from 0.9% in SynCom 8A to 12.8% in SynCom 5C) and was not significantly different between all the conditions (pairwise Wilcoxon; Figure S4A, Supporting Information). Except for SynCom 3A and SynCom 5C, the inoculated seed had more contribution to seedling microbiota compared to native seed or potting soil sources (Wilcoxon tests, Fig. 6A; Figure S4B, Supporting Information). Also, native seed microbiota had a higher contribution to seedling microbiota compared to potting soil only for SynCom 3A and SynCom 8A (pairwise Wilcoxon; Figure S4B, Supporting Information). In the control seedlings, native seed and potting soil microbiota contributed equally to the seedling microbiota (pairwise Wilcoxon; Figure S4B, Supporting Information). Overall, SynCom composition influenced the contribution of the different microbial sources driving seedling microbiota assembly.

Although seedling microbiota was mainly composed of SynCom strains, about 20% of taxa were derived from other environmental sources. This provides an opportunity to assess the role of SynCom composition on recruitment of these taxa. After removing ASVs of SynComs strains, the similarities between seedling communities were assessed with Bray–Curtis distances (Fig. 6B). Even in the absence of SynCom members, the inoculation of SynComs remained an important driver of seedling community structure (PERMANOVA, *R*^2^ = 38.76%, *P*-value = .001, Fig. 6B). Taxonomic profile of the recruited microbiota was described but showed no clear pattern (Figure S5A, Supporting Information). In seedlings derived from uninoculated seeds (control), one ASV of *Enterobacter cloacae* was highly abundant (56% of seedling microbiota; Figure S5B, Supporting Information). The relative abundance of this ASV decreased in seedling from seeds inoculated with SynComs ranging from 0.012% in SynCom 8C to 16% in SynCom 3A (Figure S5B, Supporting Information).

**Figure 7.**
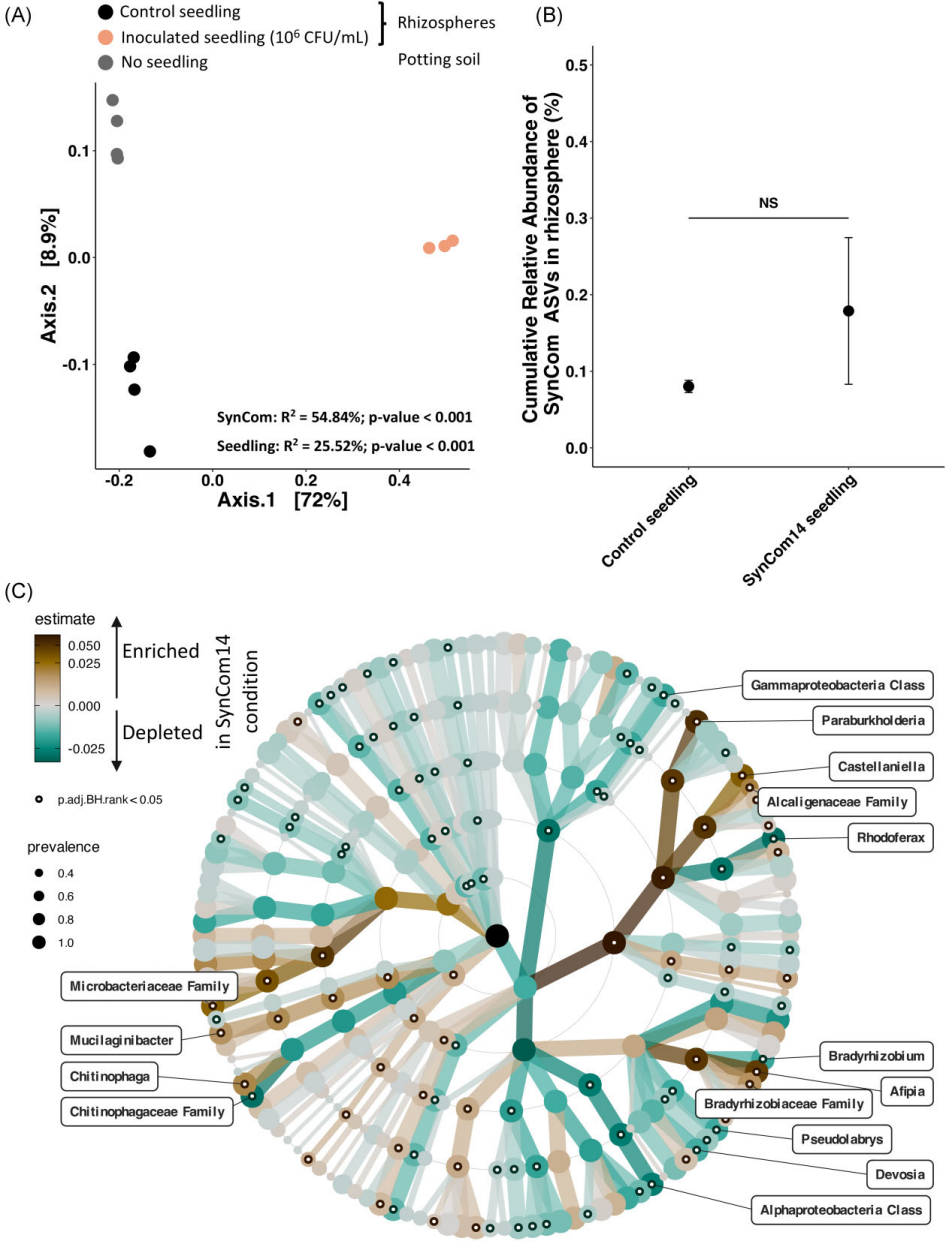
Effect of SynCom14 on rhizosphere community. (A) Potting soil bacterial community structure visualized through a PCoA ordination based on Bray–Curtis distances. The potting soil bacterial communities were studied without seedling (no seedling), with a seedling from a non-inoculated seed (control seedling), and with a seedling coming from inoculated-seed with the SynCom14 (PERMANOVA; SynCom: *R*^2^ = 54.84%, *P*-value < .001; seedling effect: *R*^2^ = 25.52%, *P*-value < .001). (B) Cumulative relative abundance of SynCom14 ASVs in rhizospheres of control and inoculated seedlings (NS: non significant Wilcoxon test). (C) Changes in the relative abundance of bacterial genera of rhizospheres of inoculated seedlings (SynCom14) or in control seedlings (not inoculated) at the different taxa levels using a linear model. Labels of the corresponding genera were plotted only if adjusted *P*-value < .05 and if estimate was below and above 0.01.

Next, we assessed the effects of SynCom inoculation on seeds on the rhizosphere microbiota. The potting soil bacterial communities were studied on day 7 without seedling (no seedling), with a seedling that had not been inoculated (control seedling), and with a seedling inoculated with one SynCom (SynCom14, Exp 2). Permanova analysis showed that 55% of the variance was explained by the inoculation while 26% was explained by the seedling presence itself (Fig. 7A). ASVs corresponding to SynCom14 members had a cumulative relative abundance of < 0.2% in the rhizospheres of control and inoculated plants (Fig. 7B). Hence, SynCom14 members did not colonize and/or persist in the surrounding soil 7 days post-inoculation. Nevertheless, the inoculation of SynCom14 led to significant differences in taxonomic composition of the rhizospheres (Fig. 7C). For instance, inoculation of SynCom14 on seeds led to an increased relative abundance of *Paraburkholderia, Castellaniella*, and *Chitinophaga* and a decreased relative abundance of *Devosia, Rhodoferax, and Pseudolabrys* compared to the control condition (Fig. 7C). Thus, SynCom14 inoculated on seed at day 0 induced modifications on seedling rhizosphere composition at day 7 without colonizing it.

## Discussion

### The use of SynCom to study ecological processes during seed and seedling microbiota assembly

Microbiota characterization on individual seeds demonstrated their low carrying capacity, their high variability in terms of diversity and composition (Chesneau et al. 2022, Simonin et al. 2022). Therefore, it is difficult to establish causality between seed microbiota composition and seedling microbiota composition. Here, we showed that common bean seed can be colonized by contrasted SynCom concentrations and compositions, validating our first hypothesis (H1). This SynCom inoculation method could be an interesting strategy for improving our understanding of seed to seedling microbiota transmission and the ecological processes involved in plant microbiota assembly (Vorholt et al. 2017). Especially, our metabarcoding approach using *gyrB* gene enables the tracking of individual strains within different niches and in different plant species (Simonin et al. 2023). Among the 36 strains selected in our study only two have the same *gyrB* ASV compared with 19 with the v4 region of 16S rRNA gene (Figure S8, Supporting Information). This microbiota engineering method enables more accurate sources-sink analysis by deeply decreasing the unknown source fraction contributing to seedling microbiota assembly in native seed communities (Rochefort et al. 2021, Kim et al. 2022). Using this method, we showed that the primary source of microorganisms for the seedling was not the potting soil but was the inoculated seed. The seed microbiota can thus be an important source of microorganisms for seedling microbiota assembly, as reported by previous studies (Johnston-Monje et al. 2021, Moroenyane et al. 2021).

Strong changes in community composition are observed during seed germination and seedling emergence (Barret et al. 2015). These microbiota shifts are mainly described as a consequence of a deep modification in plant physiology that leads to the selection of specific microorganisms (Torres-Cortés et al. 2018, Chesneau et al. 2022). Indeed, during seed germination, diverse seed exudates are secreted in the surrounding soil, which influences the microbial communities and form the spermosphere (Nelson 2018, Aziz et al. 2021). Also, neutral events such as dispersion and mass effect are expected to play a role during seedling microbiota assembly but are less described. In this context, the method exposed here is interesting to decipher the relative importance of neutral and selective processes during seed and seedling microbial community assemblies. Indeed, by manipulating the concentration of inoculum and varying the composition and richness of multiple SynComs we were able to better characterize the importance of mass effect and selection processes during seedling emergence.

### Mass effects during seed and seedling colonization

In our study model (common bean), seeds are colonized on average by 10^2^ CFU/seed (Chesneau et al. 2022), which may explain why SynCom inoculated over 10^6^ CFU/ml had completely taken over the native seed-borne community. In a coalescence framework, it means that mass effect could be more important than the priority effects that the native strains could have benefited from (Debray et al. 2022). Overall, we showed that despite the low natural carrying capacity observed on seed, common bean seeds can be colonized by different SynCom sizes, which is interesting for both theoretical and applicative frameworks.

Seed microbiota contributions to seedling microbiota under natural conditions is very variable from one study to another (Johnston-Monje et al. 2021, Rochefort et al. 2021, Walsh et al. 2021, Chesneau et al. 2022). One reason is that in natural conditions, when seed meets the soil, it also meets the soil microbiota and a diversity of possible community coalescences. Rillig et al. (2015) expose that one important parameter to predict the coalescence outcome is the mixing ratio of the two communities. In our case, we deliberately manipulated the inoculum concentration of the seed microbiota to vary this ratio. By doing so, we showed that mass effect was a key factor of the community coalescence outcome, as expected in hypothesis (H2). Indeed, SynCom contribution to seedling microbiota was correlated with inoculated seed community size. In the same way, strains relative abundance on seedlings were correlated with their relative abundance on seeds. Thus, it seems that a minimum abundance in seed is needed to be able to colonize seedlings. In the same idea, Darrasse et al. (2007) showed that to effectively infect a seedling with *Xanthomonas citri* pv. *fuscans*, a minimum of 10^3^ CFU/bean seed was needed (Darrasse et al. 2007). Arias et al. (2020) also confirmed that a minimum population size of *Xanthomonas vasicola* pv. *vasculorum* on seed was necessary to effectively colonize the plant.

To conclude, we show that mass effects drive seed and seedling microbiota assemblies (H2). Through mass effect, the seed microbiota has an advantage during seedling colonization compared to microorganisms from other environmental sources. This is a very promising result that shows that seedling microbiota can be modulated using a limited amount of inoculum on seed that is sufficient to outcompete soil microorganisms (Rocha et al. 2019).

### Selection processes during seed and seedling colonization

SynComs inoculated at the same concentration (10^7^ CFU/ml) but of different compositions show different seed colonization capacities. At the strain level, we also observed variable seed colonization capacities. It means that selection processes also occurred during seed colonization. These differences between strains mainly depend on their phylogenetic affiliation. For instance, Enterobacteriaceae are significantly enriched in seeds. These variations in seed colonization may arise from differences in the adhesive capabilities of strains, which are influenced by the secretion of prominent adhesins (Espinosa-Urgel et al. 2000, Duque et al. 2013). The selection that occurred during seed colonization seems to be the main bottleneck of seedling colonization, as we previously showed that being abundant on seed was important to colonize seedling. From an applied point of view, it could be thus interesting to develop specific seed coatings for strains with low seed colonization capacities (Rocha et al. 2019). These coatings could include binding molecules, prebiotic, and specific nutrients to maintain strain of interest on seeds.

Even if generally, the relative abundance of a strain on seed can predict its relative abundance on seedling, we also show that strains have contrasting seedling colonization capacities supporting our hypothesis (H3). These differences between strains depend on their phylogenetic groups. Torres-Cortés et al. (2018) showed that bacteria having copiotrophic strategies with rapid growth have better seedling colonization capacities via competitive exclusion processes. Consistently with our results, they showed that Enterobacteriales and Pseudomonadales were enriched during seedling emergence. In particular, we show that an *E. cloacae* was dominating the control seedling microbiota. *Enterobacter cloacae* was detected on seed but at a low relative abundance (5%), has already been described as an obligatory plant endophyte in another study (Madmony et al. 2005) and presents opportunistic characteristics with genes implied in colonization processes and copiotrophic strategy (Guérin et al. 2020, Roberts et al. 1992). These different observations suggest that redundant copiotrophic strategies of these strains increase their fitness during spermosphere formation and seedling emergence. Indeed, the multiple nutrients that are released during these events gives them clear advantages that select them.

Beyond intrinsic strain capacity to colonize seedling, we also show that biotic interactions are involved during seedling microbiota assembly. For instance, *Frigoribacterium sp* (CFBP9029) and *Microbacterium sp* (CFBP9034) have a better seedling colonization in SynCom 8B and 11A than in SynCom 5C. Interestingly, their improved seedling colonization appears in SynCom with a higher richness level. This might highlight some facilitating processes (e.g. niche expansion) from the other strains (Li et al. 2019). On the contrary, *Sphingomonas sp* (CFBP9021) is never transmitted in SynCom 11A but has a transmission rate of 75% in SynCom 5A and 8B, which might highlight some exclusion through interference (i.e. antagonism) or exploitative (i.e. niche occupation) competition (Hibbing et al. 2010). Thus, strain colonization is SynCom dependent, as previously demonstrated in other plant habitats or ecosystems (Jones et al. 2022, Simonin et al. 2023). This confirms the complexity of biotic interactions during the seedling microbiota assembly and within SynComs and it entails considering these interactions when designing SynCom for microbiota engineering. To further dissect the respective contributions of strain identity and biotic interactions, it would be pertinent to conduct inoculations with individual strains. In this way, ecological mechanisms, such as facilitation or exclusion, could potentially be more thoroughly described during the seed to seedling microbiota assembly. This knowledge could enhance the design of SynComs with increased colonization capacity and the potential to limit seed transmission of some pathogens.

Overall, multiple selection processes also occurred during seed and seedling colonization. Strains showed variable seed and seedling colonization that depend on their phylogenetic affiliation and biotic interactions within the SynCom also influence the colonization success of the different strains. More studies are needed to elucidate the relative importance of host selection, environmental filtering, and biotic interactions during seedling microbiota assembly. Also, further investigations are needed to better characterize strain transmission pathways in plant tissues and their stability during the plant development.

### Impact of seed microbiota on rhizosphere and seedling community assembly

We showed that SynComs inoculated on seed induce changes on the overall recruited communities from environmental sources (e.g. soil, native seed community, H4). Seed microbiota is expected to highly interact with soil microbiota during the spermosphere assembly which in return influences the overall rhizosphere and plant microbiota assemblies (Aziz et al. 2021, Olofintila and Noel 2023). Johnston-Monje et al. (2016) showed that most seedling rhizosphere bacteria were seed derived. On the contrary, we showed that our SynCom strains contribution to the rhizosphere is low (< 0.2%) and identical to the control. Guo et al. (2021) also showed that contribution of seed microbiota to the assembly of the rhizosphere microbiota was negligible (Guo et al. 2021). In our case, even if the SynCom14 strain contribution was low, the overall rhizosphere community assembly was modified (H4). This modification could arise from the priority effect of the inoculated strains on the initial spermosphere microbiota assembly (Aziz et al. 2021) and subsequent developing rhizosphere community. Our results are consistent with Ridout et al. (2019) showing a similar pattern with seed endophytes that influence the rhizosphere colonization of secondary symbionts through priority effect. The engineered seed communities could have changed the whole rhizosphere assembly through niche modification, biotic interactions and/or host control modification (Xu et al. 2023). For instance, Kong et al. (2021) showed that the inoculation of a plant using a specific *Bacillus amyloliquefaciens* strain could induce changes in volatile organic compounds emission that led to deep rhizosphere modification (Kong et al. 2021). In the same way, co-inoculation of *Mesorhizobium ciceri* and *Bacillus subtilis* on seed induced changes in root exudates and rhizosphere microbiota assembly of chickpea (Shcherbakova et al. 2017). Rhizosphere assembly modification could also come from biotic interactions during the coalescence between the SynCom and the potting soil communities (Rocca et al. 2021, Aziz et al. 2021). Then, because the rhizosphere is one of the main sources of microorganisms for plant microbiota (Xiong et al. 2021), it could be a factor explaining the differences observed in the recruited communities between the different SynComs. Indeed, we also showed that the extensive seedling microbiota assembly was modified by the SynCom inoculation on seeds. In this context, we can argue that multiple successive selective processes led to the extensive rhizosphere and seedling microbiota assembly changes. At first glance, the SynCom can indeed colonize seedling and rhizosphere and interact with native communities. Then, through seedling physiological modification, the entire seedling and rhizosphere niches could be modified leading to differences in microbial colonization. Overall, our study shows that seed microbiota, through priority effect, is of great interest for microbiota engineering to modulate the overall seedling and rhizosphere microbiota assemblies.

From a risk assessment point of view, the low colonization capacity of the inoculated seed microbiota into the rhizosphere could be taken as an advantage point. Indeed, the SynCom strains showed low environmental invasion capacities while still modifying the recruitment of native bacteria from the environment.

## Conclusions and prospects

We presented a simple and effective seedling microbiota engineering method using SynCom inoculation on bean seeds. The method was successful using a wide diversity of SynCom compositions (13 SynComs) and strains (36 strains) that are representative of the common bean seed microbiota. First, this method enables the modulation of seed composition and community size, even in a coalescence context with the native seed microbiota (i.e. unsterilized seeds). Then, this SynCom colonization was effective in a second coalescence event with unsterilized potting soil. SynComs contributed on average to 80% of the seedlings’ microbiota. We showed that the mass effect was the main driver of seedling microbiota colonization. Additionally, individual strains showed variable seed and seedling colonization capacities that mostly depended on their phylogenetic affiliation. Finally, through priority effects, the engineered seed microbiota modified the overall seedling and rhizosphere microbiota assemblies.

However, some questions remain unanswered, in particular on the fate of the strains in the different seedling compartments and their stability during the plant development (Abdelfattah et al. 2023). Some studies reported that seed microbiota tend to colonize preferably the aerial parts of the plants, while roots were more colonized by soil microorganisms (Moroenyane et al. 2021, Kim et al. 2022). These results are coherent with the low colonization of our strains in the rhizosphere but deserve more investigations.

Additionally, our inoculation experiments were limited to a single potting soil substrate. Walsh et al. (2021) showed that seed microbiota transmission to seedlings is influenced by the resident soil microbiota composition. Thus, it is crucial to explore diverse soils in future investigations. In this sense, Rocha et al. (2019) reported that only 25% of the seed coating experiments were conducted in field condition. Moreover, recent studies discuss the challenges of transferring laboratory-developed methods into real field conditions (Kaminsky et al. 2019, Russ et al. 2023). In this context, we are convinced that it will be necessary to employ more holistic approaches that consider both abiotic and biotic parameters of these diverse environments (Awasthi 2019). These steps are important to determine the robustness and generalizability of our method in manipulating seedling microbiota effectively under multiple field conditions.

## Supplementary Material

fiae027_Supplemental_File
