# Correction to: Seedling microbiota engineering using bacterial synthetic community inoculation on seeds

**DOI:** 10.1093/femsec/fiae065

**Published:** 2024-05-15

This is a correction to: Gontran Arnault, Coralie Marais, Anne Préveaux, Martial Briand, Anne-Sophie Poisson, Alain Sarniguet, Matthieu Barret, Marie Simonin, Seedling microbiota engineering using bacterial synthetic community inoculation on seeds, *FEMS Microbiology Ecology*, Volume 100, Issue 4, April 2024, fiae027, https://doi.org/10.1093/femsec/fiae027

In the originally published version of this manuscript the supplementary data files were incomplete and missing supplementary figures S2 and onwards. This has now been corrected.

