# Supplementary material for: Seedling microbiota engineering using bacterial synthetic community inoculation on seeds
Source: FEMS Microbiol Ecol. 2024 Mar 19;100(4):fiae027. doi: 10.1093/femsec/fiae027 (PMC10977042; doi:10.1093/femsec/fiae027)
Supplement: fiae027_Supplemental_File [file fiae027_supplemental_file.pdf]

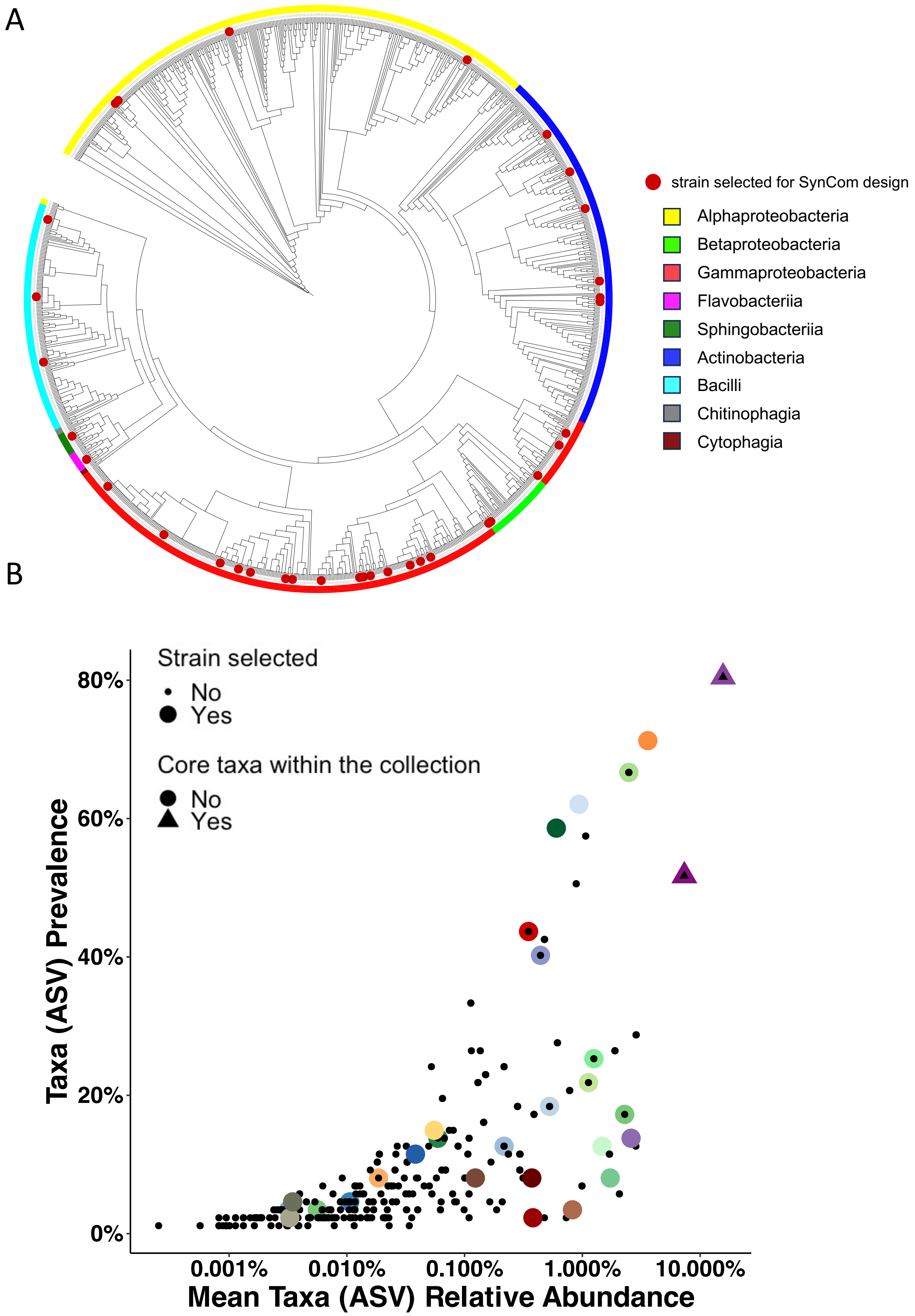

Figure S1: Strain selection for SynCom design

A) Phylogenetic tree of the 1276 strains of the collection. Phylogenetic tree was made using maximum likelihood method on *gyrB* gene. A total of 36 strains were selected (red dot). B) Strains were selected based on their prevalence and relative abundance in the common bean seed microbiota. Seven strains that were not detected in metabarcoding were also selected to include rare taxa.

Experiment 1

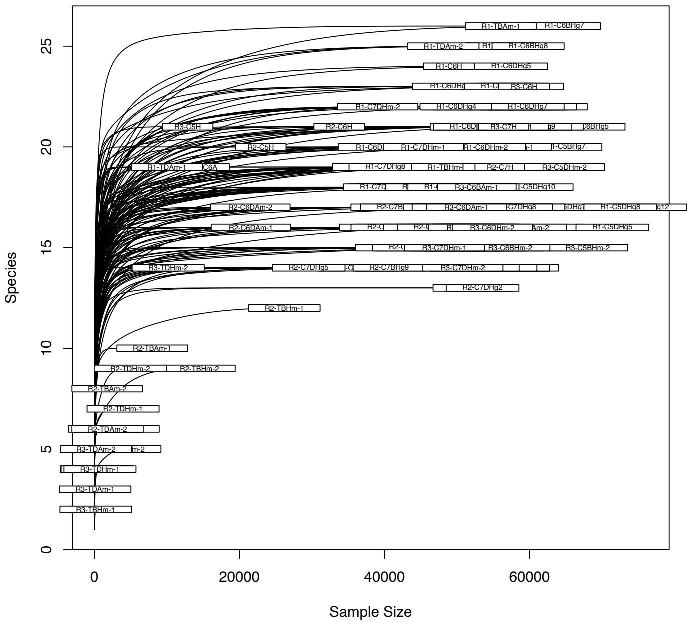

Experiment 2

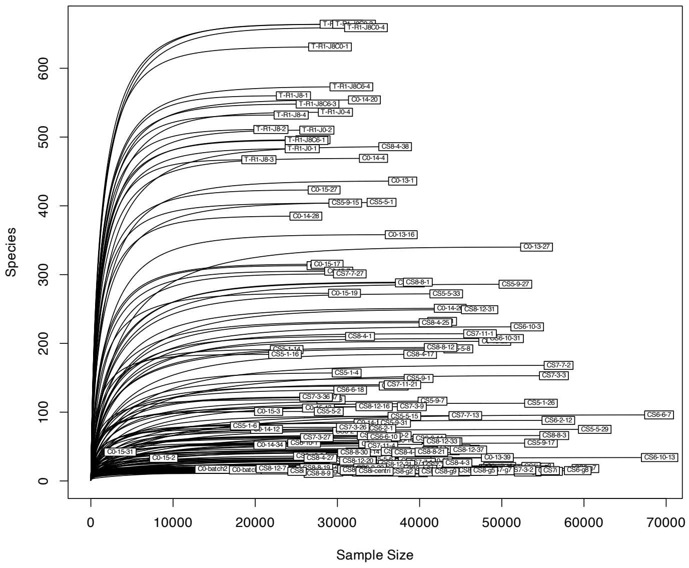

Experiment 3

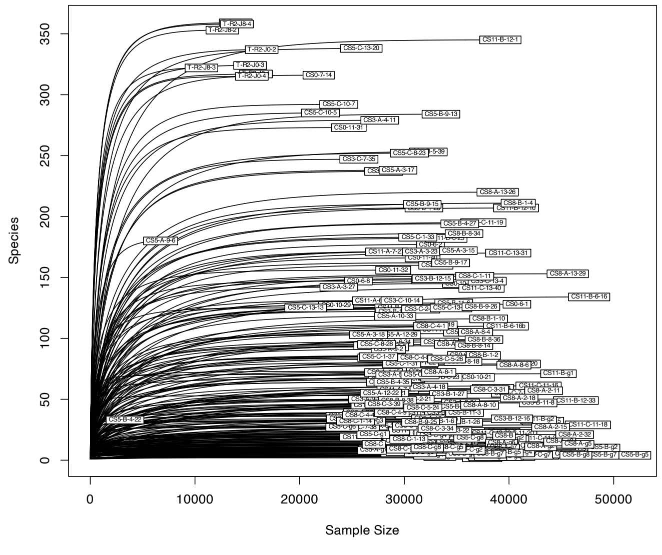

Figure S2: Rarefaction curves for the different experiments

Rarefaction curves for each sample of experiment 1, 2 and 3. For ordination analysis dataset were rarefied at 10000 reads for experiment 1. 6000 reads for experiment 2 and 10000 reads for experiment 3. For alpha diversity analysis dataset were rarefied at the minimum coverage from each experiment.

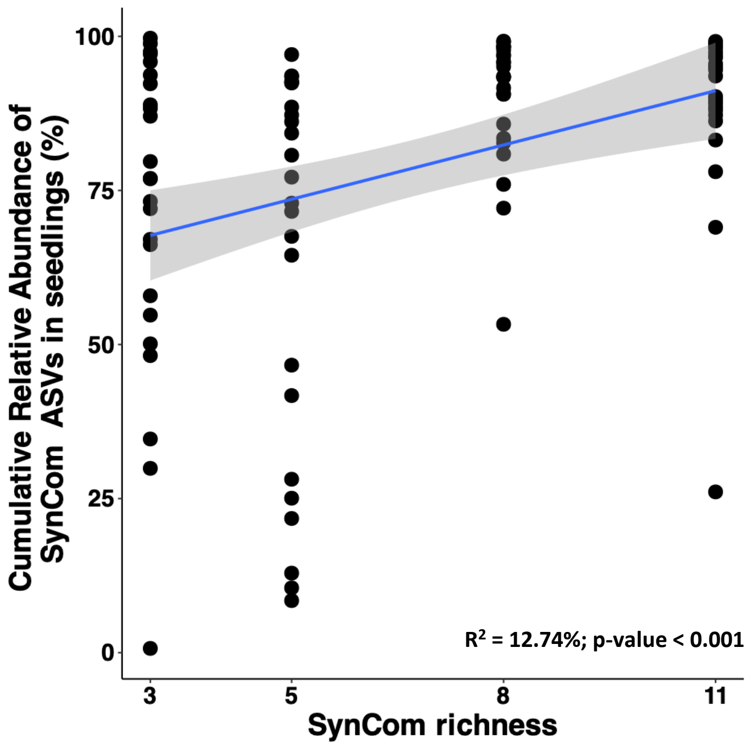

Figure S3:

Linear model between cumulative relative abundance of SynCom ASVs in seedlings and SynCom richness.

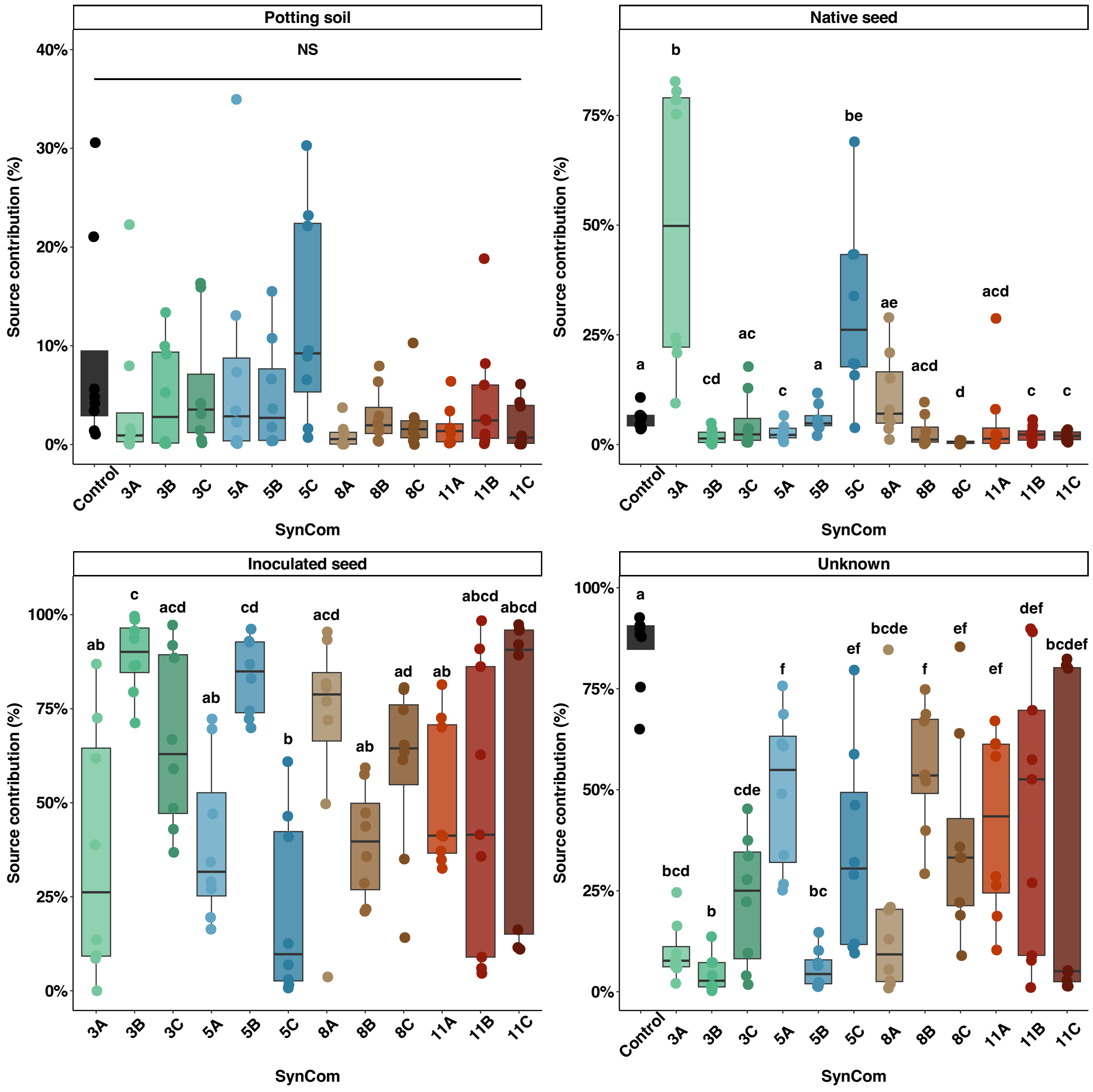

Figure S4-A:

Relative contribution of native seed microbiota, potting soil and inoculated seed, using a microbial source tracking analysis (FEAST). Native seed, inoculated seed and potting soil microbiota were considered as sources of microorganisms and seedling were considered as sink. Detailed boxplot for each source and SynCom. The different letters indicate the significantly different groups (pairwise Wilcoxon. p-value < 0.05 corrected using Benjamini-Hochberg method).

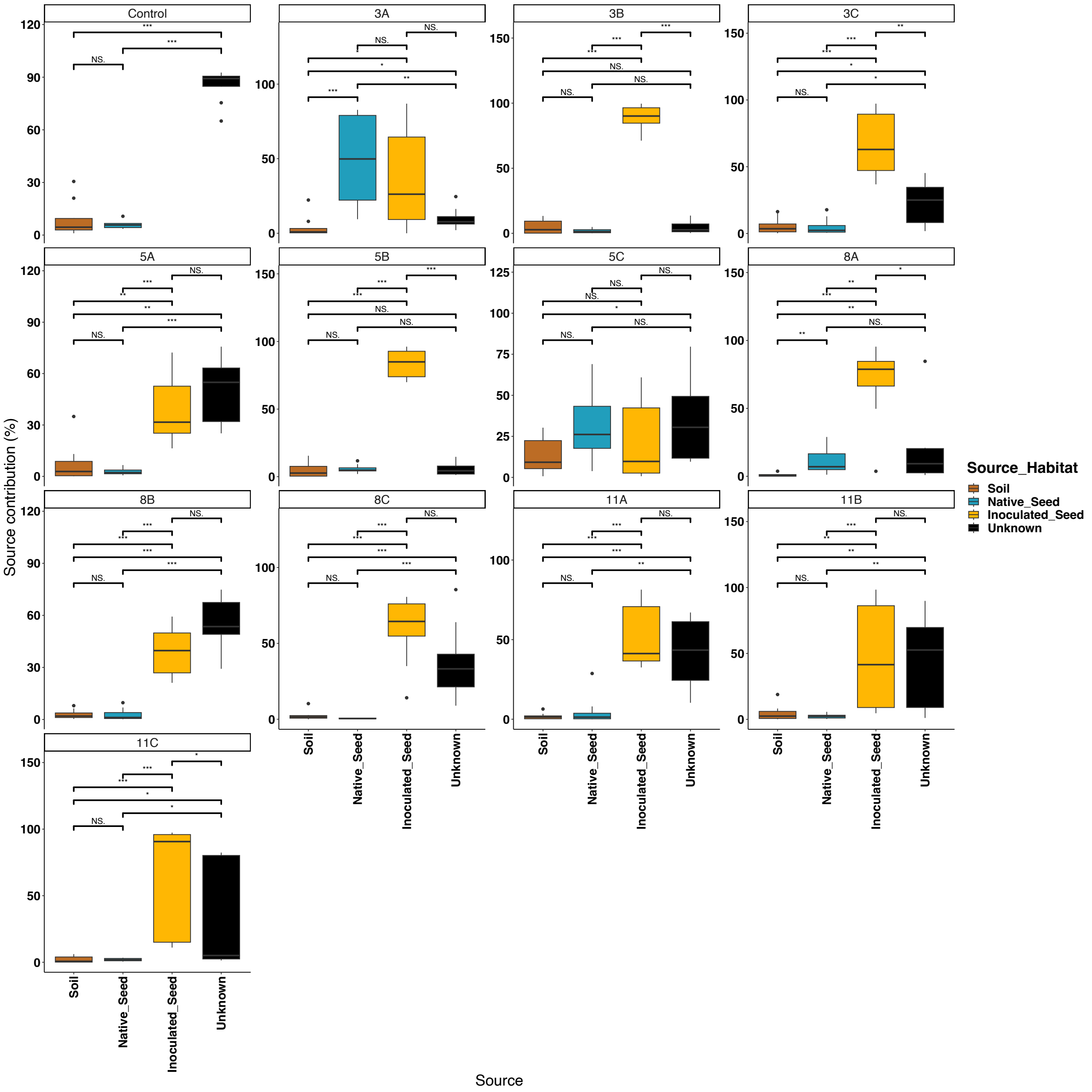

Figure S4-B:

Relative contribution of native seed microbiota, potting soil, inoculated seed and unknown source using a microbial source tracking analysis (FEAST). Native seed, inoculated seed and potting soil microbiota were considered as sources of microorganisms and seedling were considered as sink. Detailed boxplot for each SynCom. Pairwise Wilcoxon; NS.: not significant; *: p-value < 0.05; **: p-value < 0.01; ***: p-value < 0.001; p-values were corrected using Benjamini-Hochberg method).

A

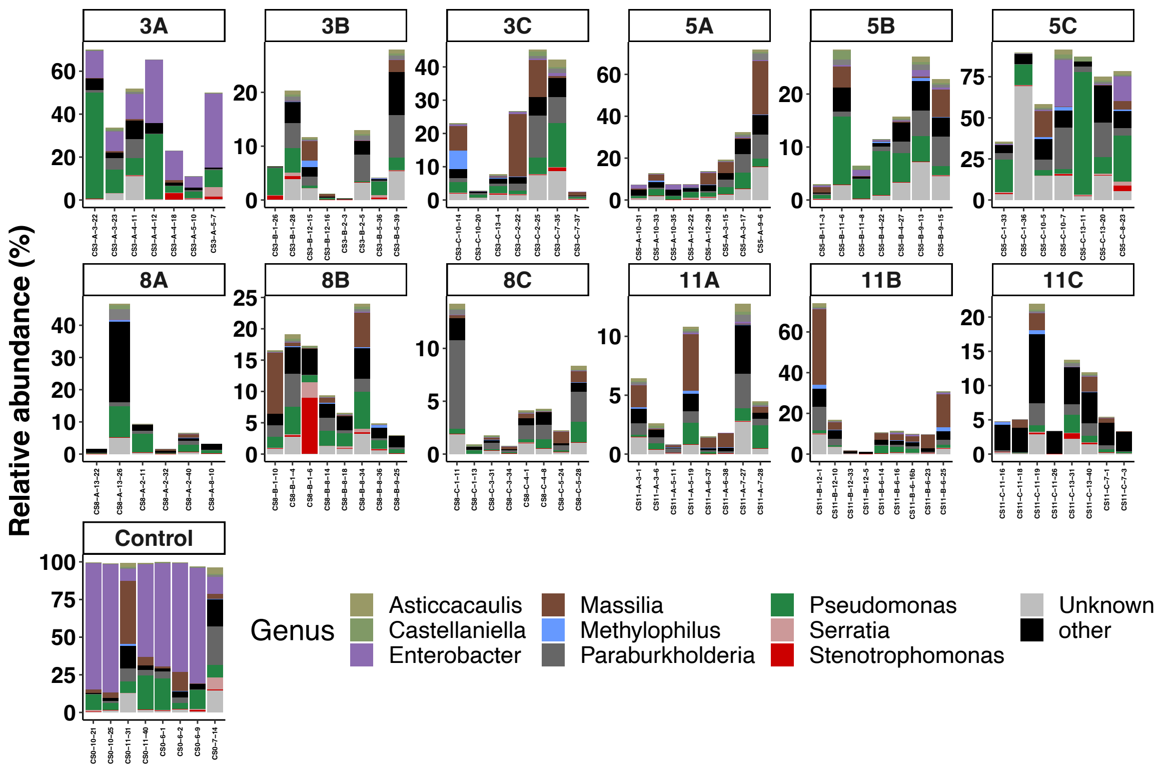

B

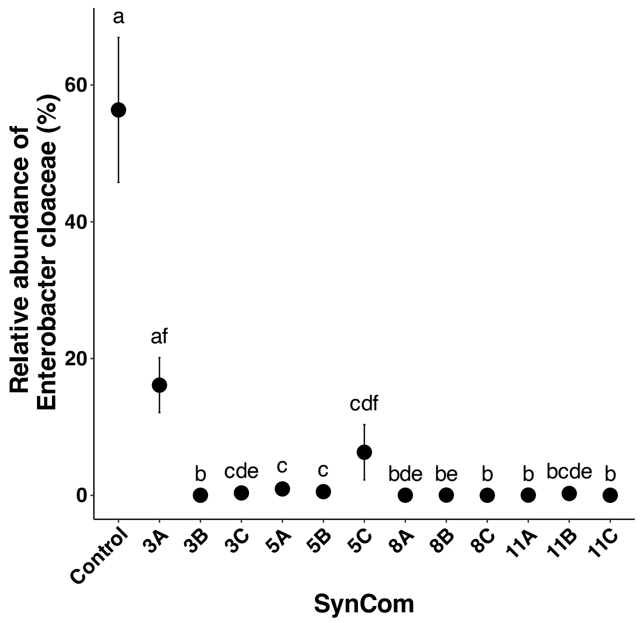

Figure S5:

A) Taxonomic profiles of the seedlings in the different SynComs of experiment 3. Each stacked bar represents a sample. ASVs of the SynCom strains were removed to plot only the recruited fraction from the environment and the native seeds. ASVs were agglomerated at the genus level and genus that represented less than 0.1% were put in the ‘other’ category (black). B) Focus on *Enterobacter cloaceae* relative abundance in seedlings of experiment 3 (the different letters indicate the statistically different groups (p-value < 0.05) using pairwise Wilcoxon test. p-value were corrected using Benjamini-Hochberg correction).

A

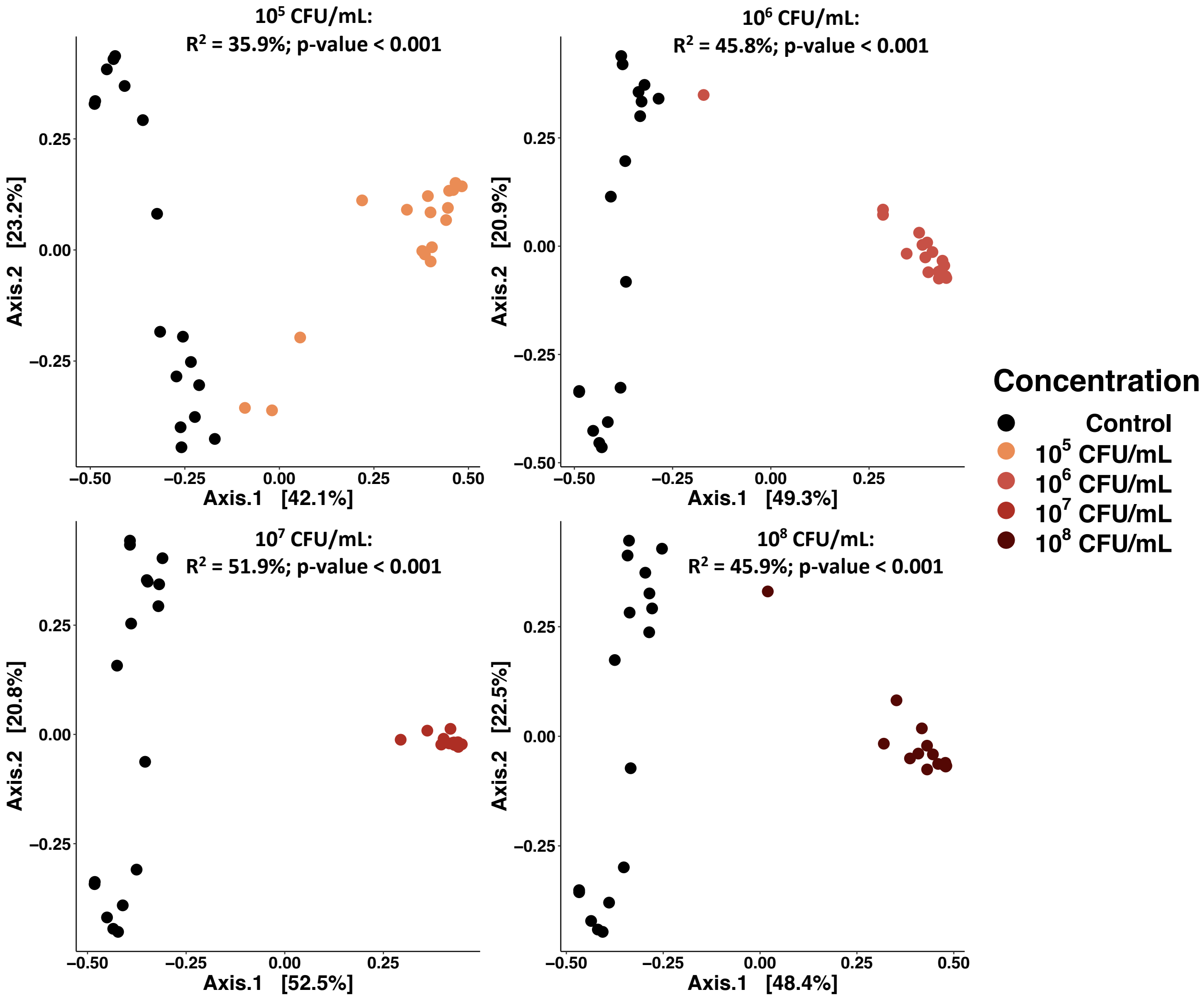

B

pairs	Df	SumsOfSqs	F.Model	R2	p.value	p.adjusted (BH)	significance
0 vs 5	1	3.72	17.906	0.359	0.001	0.002	*
0 vs 6	1	5.00	27.836	0.458	0.001	0.002	*
0 vs 7	1	5.39	33.469	0.519	0.001	0.002	*
0 vs 8	1	4.62	25.502	0.459	0.001	0.002	*
5 vs 6	1	0.34	2.631	0.074	0.037	0.056	
5 vs 7	1	0.63	5.844	0.159	0.001	0.002	*
5 vs 8	1	0.34	2.674	0.082	0.039	0.056	
6 vs 7	1	0.10	1.226	0.037	0.250	0.278	
6 vs 8	1	0.08	0.812	0.026	0.540	0.540	
7 vs 8	1	0.12	1.599	0.052	0.145	0.181	

Figure S6:

A) Influence of SynCom14 concentration on seedling bacterial community structure visualized through a PCoA ordination based on Bray-Curtis distances (PERMANOVA tests are indicated in each panel). B) Summary table of the pairwise PERMANOVA for seedlings of experiment 3. 0: Control; 5: inoculated with SynCom14 at 10^5 CFU/mL; 6: 10^6 CFU/mL, 7: 10^7 CFU/mL, 8: 10^8 CFU/mL.

pairs	Df	SumsOfSqs	F.Model	R2	p.value	p.adjusted (BH)	significance
0 vs 11A	1	3.00	27.68	0.664	0.002	0.002	*
0 vs 11B	1	2.85	23.11	0.606	0.001	0.001	*
0 vs 11C	1	3.24	40.63	0.744	0.001	0.001	*
0 vs 3A	1	1.25	7.76	0.374	0.004	0.004	*
0 vs 3B	1	3.05	37.20	0.727	0.001	0.001	*
0 vs 3C	1	2.55	23.72	0.646	0.001	0.001	*
0 vs 5A	1	2.67	23.99	0.649	0.001	0.001	*
0 vs 5B	1	2.42	24.96	0.675	0.001	0.001	*
0 vs 5C	1	1.04	4.84	0.271	0.004	0.004	*
0 vs 8A	1	2.39	16.90	0.585	0.001	0.001	*
0 vs 8B	1	2.95	26.86	0.657	0.001	0.001	*
0 vs 8C	1	3.03	28.29	0.669	0.001	0.001	*
11A vs 11B	1	3.44	40.89	0.732	0.001	0.001	*
11A vs 11C	1	3.66	97.31	0.874	0.001	0.001	*
11A vs 3A	1	2.16	18.62	0.589	0.002	0.002	*
11A vs 3B	1	1.05	26.46	0.654	0.001	0.001	*
11A vs 3C	1	3.18	51.17	0.797	0.001	0.001	*
11A vs 5A	1	2.80	42.48	0.766	0.002	0.002	*
11A vs 5B	1	3.00	63.11	0.840	0.001	0.001	*
11A vs 5C	1	1.99	11.74	0.474	0.002	0.002	*
11A vs 8A	1	2.79	30.18	0.716	0.001	0.001	*
11A vs 8B	1	2.87	42.26	0.751	0.001	0.001	*
11A vs 8C	1	0.36	5.57	0.284	0.002	0.002	*
11B vs 11C	1	3.65	63.54	0.809	0.001	0.001	*
11B vs 3A	1	2.77	21.05	0.601	0.001	0.001	*
11B vs 3B	1	3.58	60.25	0.801	0.001	0.001	*
11B vs 3C	1	3.08	37.69	0.729	0.001	0.001	*
11B vs 5A	1	3.15	36.97	0.725	0.001	0.001	*
11B vs 5B	1	2.90	41.61	0.762	0.001	0.001	*
11B vs 5C	1	2.18	11.98	0.461	0.001	0.001	*
11B vs 8A	1	1.86	16.78	0.563	0.001	0.001	*
11B vs 8B	1	3.04	35.53	0.703	0.002	0.002	*
11B vs 8C	1	1.99	24.02	0.616	0.001	0.001	*
11C vs 3A	1	2.99	35.19	0.730	0.001	0.001	*
11C vs 3B	1	3.81	343.49	0.961	0.001	0.001	*
11C vs 3C	1	2.76	87.99	0.871	0.001	0.001	*
11C vs 5A	1	3.11	88.49	0.872	0.001	0.001	*
11C vs 5B	1	2.89	202.86	0.944	0.001	0.001	*
11C vs 5C	1	2.49	17.90	0.579	0.001	0.001	*
11C vs 8A	1	2.62	44.30	0.787	0.002	0.002	*
11C vs 8B	1	3.31	84.48	0.858	0.001	0.001	*
11C vs 8C	1	3.67	101.29	0.879	0.003	0.003	*
3A vs 3B	1	2.90	33.24	0.719	0.002	0.002	*
3A vs 3C	1	2.52	21.82	0.645	0.001	0.001	*
3A vs 5A	1	2.54	21.21	0.639	0.001	0.001	*
3A vs 5B	1	2.37	22.69	0.674	0.001	0.001	*
3A vs 5C	1	1.27	5.46	0.313	0.001	0.001	*
3A vs 8A	1	1.96	12.77	0.537	0.002	0.002	*
3A vs 8B	1	1.76	14.99	0.536	0.001	0.001	*
3A vs 8C	1	2.80	24.44	0.653	0.001	0.001	*
3B vs 3C	1	3.26	96.74	0.882	0.001	0.001	*
3B vs 5A	1	3.30	88.11	0.871	0.001	0.001	*
3B vs 5B	1	3.07	183.72	0.939	0.002	0.002	*
3B vs 5C	1	2.01	14.19	0.522	0.001	0.001	*
3B vs 8A	1	2.92	47.38	0.798	0.001	0.001	*
3B vs 8B	1	3.54	85.71	0.860	0.002	0.002	*
3B vs 8C	1	1.01	26.24	0.652	0.001	0.001	*
3C vs 5A	1	2.05	33.28	0.735	0.001	0.001	*
3C vs 5B	1	0.18	4.26	0.279	0.01	0.010	*
3C vs 5C	1	1.83	10.53	0.467	0.001	0.001	*
3C vs 8A	1	0.47	5.19	0.320	0.002	0.002	*
3C vs 8B	1	2.25	35.21	0.730	0.001	0.001	*
3C vs 8C	1	3.20	52.72	0.802	0.001	0.001	*
5A vs 5B	1	2.77	60.59	0.846	0.001	0.001	*
5A vs 5C	1	2.10	11.75	0.495	0.001	0.001	*
5A vs 8A	1	2.61	27.62	0.715	0.001	0.001	*
5A vs 8B	1	1.39	20.58	0.613	0.001	0.001	*
5A vs 8C	1	2.98	46.20	0.780	0.001	0.001	*
5B vs 5C	1	1.77	10.50	0.488	0.001	0.001	*
5B vs 8A	1	0.34	4.53	0.312	0.002	0.002	*
5B vs 8B	1	2.95	59.62	0.832	0.001	0.001	*
5B vs 8C	1	3.03	65.86	0.846	0.002	0.002	*
5C vs 8A	1	1.76	8.08	0.423	0.001	0.001	*
5C vs 8B	1	2.23	12.98	0.500	0.001	0.001	*
5C vs 8C	1	2.04	12.12	0.482	0.002	0.002	*
8A vs 8B	1	2.72	28.86	0.706	0.001	0.001	*
8A vs 8C	1	2.81	30.93	0.720	0.001	0.001	*
8B vs 8C	1	3.42	51.54	0.786	0.001	0.001	*

Figure S7:

Summary table of the pairwise PERMANOVA for seedlings of experiment 3. p-values were adjusted using Benjamini-Hochberg correction.

Strain	md5 16S (V4)	16S (V4)	Shared 16S (V4)	Shared *gyrB*
Pseudomonas fluorescens subgroup (8992)	f8aeec3c1ff0f4e7e1c829e4db0618d9	TACAGAGGGTGCAAGCGTTAATCGGAATTACTGGGCGTAAAGCGCGCGTAGGTGGTTTGTTAAGTTGGATGTGAAATCCCCGGGCTCAACCTGGGAACTGCATTCAAAACTGACTGACTAGAGTATGGTAGAGGGTGGTGGAATTTCCTGTGTAGCGGTGAAATGCGTAGATATAGGAAGGAACACCAGTGGCGAAGGCGACCACCTGGACTGATACTGACACTGAGGTGCGAAAGCGTGGGGAGC	No	No
Pseudomonas koreensis subgroup (9003)	0f18144d308ada95632ab5193d92073f	TACAGAGGGTGCAAGCGTTAATCGGAATTACTGGGCGTAAAGCGCGCGTAGGTGGTTTGTTAAGTTGGATGTGAAATCCCCGGGCTCAACCTGGGAACTGCATCCAAAACTGGCAAGCTAGAGTATGGTAGAGGGTGGTGGAATTTCCTGTGTAGCGGTGAAATGCGTAGATATAGGAAGGAACACCAGTGGCGAAGGCGACCACCTGGACTGATACTGACACTGAGGTGCGAAAGCGTGGGGAGC	No	No
Pseudomonas syringae (8979)	43fddf1528d4a98928fd8c3a8ac23bfd	TACAGAGGGTGCAAGCGTTAATCGGAATTACTGGGCGTAAAGCGCGCGTAGGTGGTTTGTTAAGTTGAATGTGAAATCCCCGGGCTCAACCTGGGAACTGCATCCAAAACTGGCAAGCTAGAGTATGGTAGAGGGTGGTGGAATTTCCTGTGTAGCGGTGAAATGCGTAGATATAGGAAGGAACACCAGTGGCGAAGGCGACCACCTGGACTGATACTGACACTGAGGTGCGAAAGCGTGGGGAGC	No	No
Pseudomonas viridiflava (8985)	**b4289ab0a9a00034c1fbce477b2c4ded**	**TACAGAGGGTGCAAGCGTTAATCGGAATTACTGGGCGTAAAGCGCGCGTAGGTGGTTTGTTAAGTTGAATGTGAAATCCCCGGGCTCAACCTGGGAACTGCATCCAAAACTGGCAAGCTAGAGTAGGGCAGAGGGTGGTGGAATTTCCTGTGTAGCGGTGAAATGCGTAGATATAGGAAGGAACACCAGTGGCGAAGGCGACCACCTGGGCTCATACTGACACTGAGGTGCGAAAGCGTGGGGAGCAAACAGG**	Yes	No
Pseudomonas viridiflava (8991)			Yes	Yes
Pseudomonas viridiflava (8983)			Yes	Yes
Pseudomonas putida group (8984)			Yes	No
Pseudomonas coleopterorum (8982)			Yes	No
Pseudomonas coleopterorum (8977)			Yes	No
Chryseobacterium sp (8996)	e61a7bc42a30f0ca4f170b5e3f6cad0c	TACGGAGGGTGCAAGCGTTATCCGGATTTATTGGGTTTAAAGGGTCCGTAGGCGGATCTGTAAGTCAGTGGTGAAATCTCATAGCTTAACTATGAAACTGCCATTGATACTGCAGGTCTTGAGTAAAGTAGAAGTGGCTGGAATAAGTAGTGTAGCGGTGAAATGCATAGATATTACTTAGAACACCAATTGCGAAGGCAGGTCACTATGTTTTAACTGACGCTGATGGACGAAAGCGTGGGGAGCGA	No	No
Pedobacter sp (9032)	6ee76f7c45c83dc4a6f2340844478fd2	TACGGAGGATCCAAGCGTTATCCGGATTTATTGGGTTTAAAGGGTGCGTAGGCGGCCCGTTAAGTCAGGGGTGAAAGACGGTAGCTCAACTATCGCAGTGCCTTTGATACTGACGGGCTTGAATGAACTAGAGGTAGGCGGAATGAGACAAGTAGCGGTGAAATGCATAGATATGTCTCAGAACACCGATTGCGAAGGCAGCTTACTATGGTTTAATTGACGCTGAGGCACGAAAGCGTGGGGATCA	No	No
Bacillus sp (9009)	7d88cb85b80e0024b54a61e7a6da3bf0	TACGTAGGTGGCAAGCGTTGTCCGGAATTATTGGGCGTAAAGCGCGCGCAGGTGGTTCCTTAAGTCTGATGTGAAAGCCCACGGCTCAACCGTGGAGGGTCATTGGAAACTGGGGAACTTGAGTGCAGAAGAGGAAAGTGGAATTCCAAGTGTAGCGGTGAAATGCGTAGAGATTTGGAGGAACACCAGTGGCGAAGGCGACTTTCTGGTCTGTAACTGACACTGAGGCGCGAAAGCGTGGGGAGC	No	No
Bacillus megaterium (9010)	83e948f389f9d97b0c975afc880989e8	TACGTAGGTGGCAAGCGTTATCCGGAATTATTGGGCGTAAAGCGCGCGCAGGCGGTTTCTTAAGTCTGATGTGAAAGCCCACGGCTCAACCGTGGAGGGTCATTGGAAACTGGGGAACTTGAGTGCAGAAGAGAAAAGCGGAATTCCACGTGTAGCGGTGAAATGCGTAGAGATGTGGAGGAACACCAGTGGCGAAGGCGGCTTTTTGGTCTGTAACTGACGCTGAGGCGCGAAAGCGTGGGGAGC	No	No
Bacillus thuringiensis (9014)	bdf8a26094624622d68509a87fa75ba7	TACGTAGGTGGCAAGCGTTATCCGGAATTATTGGGCGTAAAGCGCGCGCAGGTGGTTTCTTAAGTCTGATGTGAAAGCCCACGGCTCAACCGTGGAGGGTCATTGGAAACTGGGAGACTTGAGTGCAGAAGAGGAAAGTGGAATTCCATGTGTAGCGGTGAAATGCGTAGAGATATGGAGGAACACCAGTGGCGAAGGCGACTTTCTGGTCTGTAACTGACACTGAGGCGCGAAAGCGTGGGGAGC	No	No
Pantoea agglomerans (8989)	**cc761daf51f27c423da57f3f1f0ff5cc**	**TACGGAGGGTGCAAGCGTTAATCGGAATTACTGGGCGTAAAGCGCACGCAGGCGGTCTGTTAAGTCAGATGTGAAATCCCCGGGCTTAACCTGGGAACTGCATTTGAAACTGGCAGGCTTGAGTCTTGTAGAGGGGGGTAGAATTCCAGGTGTAGCGGTGAAATGCGTAGAGATCTGGAGGAATACCGGTGGCGAAGGCGGCCCCCTGGACAAAGACTGACGCTCAGGTGCGAAAGCGTGGGGAGCAAACAGG**	Yes	No
Pantoea agglomerans (8988)			Yes	No
Enterobacter sp (8995)	3b94c19ef326eea0918e1c3d244c999c	TACGGAGGGTGCAAGCGTTAATCGGAATTACTGGGCGTAAAGCGCACGCAGGCGGTCTGTCAAGTCGGATGTGAAATCCCCGGGCTTAACCTGGGAACTGCATTCGAAACTGGCAGGCTAGAGTCTTGTAGAGGGGGGTAGAATTCCAGGTGTAGCGGTGAAATGCGTAGAGATCTGGAGGAATACCGGTGGCGAAGGCGGCCCCCTGGACAAAGACTGACGCTCAGGTGCGAAAGCGTGGGGAG	No	No
Kosakonia sp (8986)	a0901407705992dc213ac509ede97d47	TACGGAGGGTGCAAGCGTTAATCGGAATTACTGGGCGTAAAGCGCACGCAGGCGGTCTGTCAAGTCGGATGTGAAATCCCCGGGCTCAACCTGGGAACTGCATCCGAAACTGGCAGGCTTGAGTCTCGTAGAGGGGGGTAGAATTCCAGGTGTAGCGGTGAAATGCGTAGAGATCTGGAGGAATACCGGTGGCGAAGGCGGCCCCCTGGACGAAGACTGACGCTCAGGTGCGAAAGCGTGGGGAG	No	No
Siccibacter turicensis (8990)	**945184b6386c192c0066e0a98a154780**	**TACGGAGGGTGCAAGCGTTAATCGGAATTACTGGGCGTAAAGCGCACGCAGGCGGTCTGTCAAGTCGGATGTGAAATCCCCGGGCTCAACCTGGGAACTGCATTCGAAACTGGCAGGCTAGAGTCTTGTAGAGGGGGGTAGAATTCCAGGTGTAGCGGTGAAATGCGTAGAGATCTGGAGGAATACCGGTGGCGAAGGCGGCCCCCTGGACAAAGACTGACGCTCAGGTGCGAAAGCGTGGGGAGCAAACAGG**	Yes	No
Lelliottia sp (8978)			Yes	No
Leclercia sp (8987)			Yes	No
Massilia sp (9012)	**4608fae4bbc9964cdd17af8782f2155e**	**TACGTAGGGTGCAAGCGTTAATCGGAATTACTGGGCGTAAAGCGTGCGCAGGCGGTTTTGTAAGTCTGTCGTGAAATCCCCGGGCTCAACCTGGGAATTGCGATGGAGACTGCAAGGCTAGAATCTGGCAGAGGGGGGTAGAATTCCACGTGTAGCAGTGAAATGCGTAGAGATGTGGAGGAACACCGATGGCGAAGGCAGCCCCCTGGGTCAAGATTGACGCTCATGCACGAAAGCGTGGGGAGCAAACAGG**	Yes	No
Massilia sp (9026)			Yes	No
Stenotrophomonas sp (8980)	fd49e69f94c6e1d27fa06bfa3ee94591	TACGAAGGGTGCAAGCGTTACTCGGAATTACTGGGCGTAAAGCGTGCGTAGGTGGTTGTTTAAGTCTGTTGTGAAAGCCCTGGGCTCAACCTGGGAACTGCAGTGGAAACTGGATGACTAGAGTGTGGTAGAGGGTAGCGGAATTCCCGGTGTAGCAGTGAAATGCGTAGAGATCGGGAGGAACATCCATGGCGAAGGCAGCTACCTGGACCAACACTGACACTGAGGCACGAAAGCGTGGGGAGC	No	No
Stenotrophomonas sp (8994)	**496ecde24f9ab698992413d3d4f04b5f**	**TACGAAGGGTGCAAGCGTTACTCGGAATTACTGGGCGTAAAGCGTGCGTAGGTGGTTGTTTAAGTCTGTTGTGAAAGCCCTGGGCTCAACCTGGGAATTGCAGTGGATACTGGGCGACTAGAGTGTGGTAGAGGGTAGTGGAATTCCCGGTGTAGCAGTGAAATGCGTAGAGATCGGGAGGAACATCCATGGCGAAGGCAGCTACCTGGACCAACACTGACACTGAGGCACGAAAGCGTGGGGAGCAAACAGG**	Yes	No
Stenotrophomonas rhizophila (9006)			Yes	No
Rhizobium sp (9020)	e1c7d97fe13e9127d225d76a1feb8c78	TACGAAGGGGGCTAGCGTTGTTCGGAATTACTGGGCGTAAAGCGCACGTAGGCGGATATTTAAGTCAGGGGTGAAATCCCAGAGCTCAACTCTGGAACTGCCTTTGATACTGGGTATCTTGAGTATGGAAGAGGTAAGTGGAATTGCGAGTGTAGAGGTGAAATTCGTAGATATTCGCAGGAACACCAGTGGCGAAGGCGGCTTACTGGTCCATTACTGACGCTGAGGTGCGAAAGCGTGGGGAGCA	No	No
Sphingomonas sp (8993)	10afda2baef44de4c584a6641de399b1	TACGGAGGGGGCTAGCGTTGTTCGGAATTACTGGGCGTAAAGCGCACGTAGGCGGCTTTGTAAGTCAGAGGTGAAAGCCTGGAGCTCAACTCCAGAACTGCCTTTGAGACTGCATCGCTTGAATCCAGGAGAGGTCAGTGGAATTCCGAGTGTAGAGGTGAAATTCGTAGATATTCGGAAGAACACCAGTGGCGAAGGCGGCTGACTGGACTGGTATTGACGCTGAGGTGCGAAAGCGTGGGGAGCA	No	No
Sphingomonas sp (9021)	**9ac3bb1d7dedb08a012692a6f536b5af**	**TACGGAGGGAGCTAGCGTTATTCGGAATTACTGGGCGTAAAGCGCACGTAGGCGGCTTTGTAAGTAAGAGGTGAAAGCCCAGAGCTCAACTCTGGAATTGCCTTTTAGACTGCATCGCTTGAATCATGGAGAGGTCAGTGGAATTCCGAGTGTAGAGGTGAAATTCGTAGATATTCGGAAGAACACCAGTGGCGAAGGCGGCTGACTGGACATGTATTGACGCTGAGGTGCGAAAGCGTGGGGAGCAAACAGG**	Yes	No
Sphingomonas sp (9019)			Yes	No
Microbacterium sp (9023)	9fe4c3dfc721f87da8ff9661f9600667	TACGTAGGGCGCAAGCGTTATCCGGAATTATTGGGCGTAAAGAGCTCGTAGGCGGTTTGTCGCGTCTGCTGTGAAATCCGGAGGCTCAACCTCCGGCCTGCAGTGGGTACGGGCAGACTAGAGTGCGGTAGGGGAGATTGGAATTCCTGGTGTAGCGGTGGAATGCGCAGATATCAGGAGGAACACCGATGGCGAAGGCAGATCTCTGGGCCGTAACTGACGCTGAGGAGCGAAAGGGTGGGGAG	No	No
Microbacterium sp (9034)	73373d2d94551b3c53c3e01b6a02aeb3	TACGTAGGGCGCAAGCGTTATCCGGAATTATTGGGCGTAAAGAGCTCGTAGGCGGTTTGTCGCGTCTGCTGTGAAATCCCGAGGCTCAACCTCGGGTCTGCAGTGGGTACGGGCAGACTAGAGTGCGGTAGGGGAGATTGGAATTCCTGGTGTAGCGGTGGAATGCGCAGATATCAGGAGGAACACCGATGGCGAAGGCAGATCTCTGGGCCGTAACTGACGCTGAGGAGCGAAAGGGTGGGGAG	No	No
Rathayibacter festucae (9033)	7ae808cd4ae2caf0e35da0589ee28745	TACGTAGGGTGCAAGCGTTGTCCGGAATTATTGGGCGTAAAGAGCTCGTAGGCGGTTTGTCGCGTCTGCTGTGAAAACCCGAGGCTCAACCTCGGGCCTGCAGTGGGTACGGGCAGACTAGAGTGCGGTAGGGGAGAATGGAATTCCTGGTGTAGCGGTGGAATGCGCAGATATCAGGAGGAACACCGATGGCGAAGGCAGTTCTCTGGGCCGTTACTGACGCTGAGGAGCGAAAGCGTGGGGAG	No	No
Curtobacterium sp (9011)	497a26126c9e726c768bdec79e8c4e9b	TACGTAGGGTGCAAGCGTTGTCCGGAATTATTGGGCGTAAAGAGCTCGTAGGCGGTTTGTCGCGTCTGCTGTGAAATCCCGAGGCTCAACCTCGGGCTTGCAGTGGGTACGGGCAGACTAGAGTGCGGTAGGGGAGATTGGAATTCCTGGTGTAGCGGTGGAATGCGCAGATATCAGGAGGAACACCGATGGCGAAGGCAGATCTCTGGGCCGTAACTGACGCTGAGGAGCGAAAGCATGGGGAG	No	No
Frigoribacterium sp (9030)	**453cf07d3e96e304f23fbde7b355fe70**	**TACGTAGGGTGCAAGCGTTGTCCGGAATTATTGGGCGTAAAGAGCTCGTAGGCGGTTTGTCGCGTCTGCTGTGAAATCTGGGGGCTCAACCCCCAGCCTGCAGTGGGTACGGGCAGACTAGAGTGCGGTAGGGGAGATTGGAATTCCTGGTGTAGCGGTGGAATGCGCAGATATCAGGAGGAACACCGATGGCGAAGGCAGATCTCTGGGCCGTAACTGACGCTGAGGAGCGAAAGCATGGGGAGCGAACAGG**	Yes	No
Frigoribacterium sp (9029)			Yes	No

Figure S8: overview of 16S V4 sequences of the 36 strains

Comparison of the traceability of the strains using the *gyrB* gene *versus* 16S (V4 region).

2 strains are sharing the same *gyrB* sequence while 19 strains are sharing 16S V4 sequence.

Sequences were extracted using in_silico_PCR.pl (https://github.com/egonozer/in_silico_pcr) using these primer sequences:

16S: GTGCCAGCMGCCGCGGTAA   GGACTACHVGGGTWTCTAAT

gyrB: MGNCCNGSNATGTAYATHGG   ACNCCRTGNARDCCDCCNGA

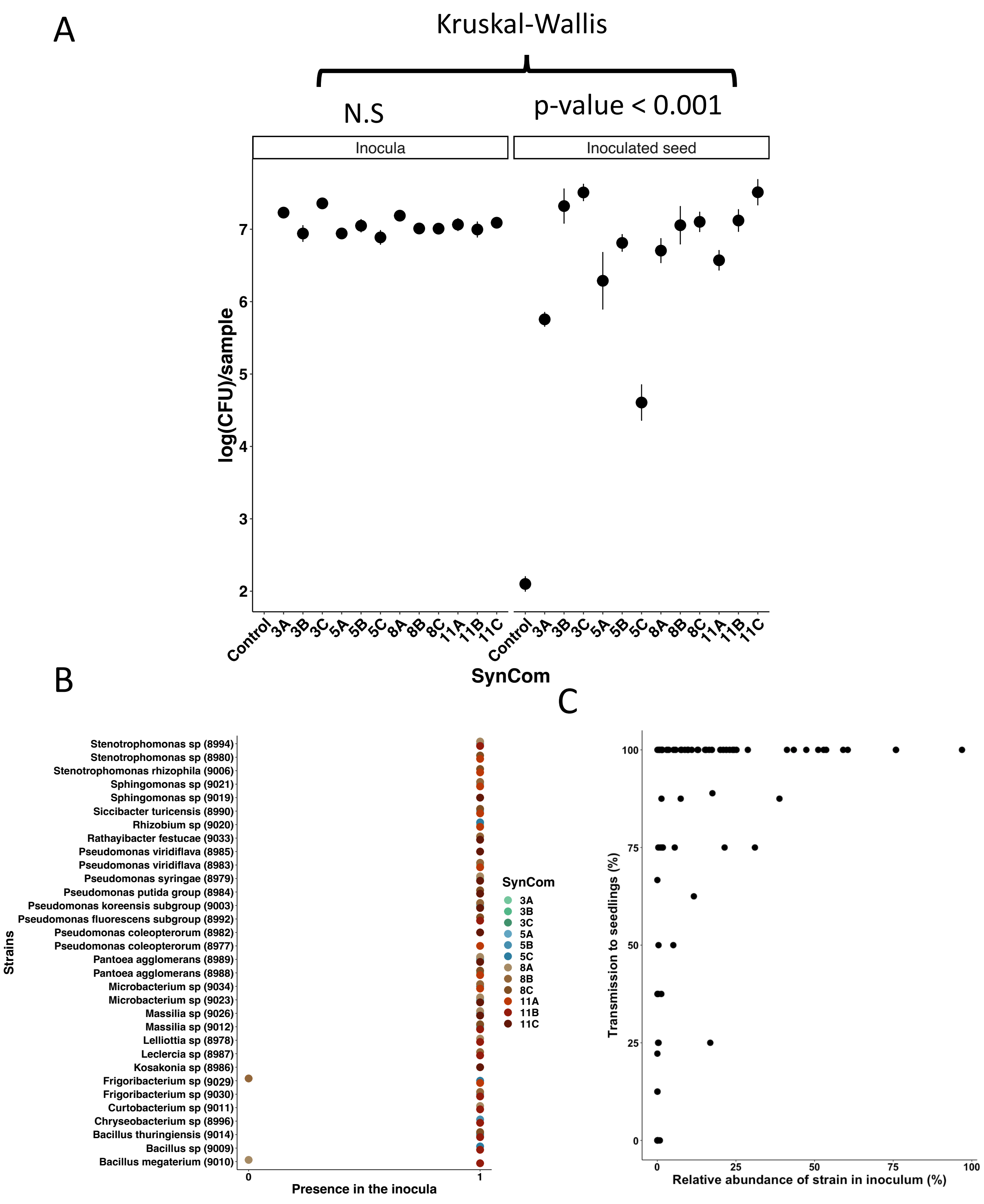

Figure S9:

A) Community sizes (log(CFU)/sample) of the inocula and the post-inoculated seeds from experiment 3.

Kruskal-Wallis tests showed non significant difference between inocula community sizes (p-value = 0.123) while inoculated seeds presented significantly different community sizes (p-value < 0.001).

B) Presence detection of each strain within the inocula of the given SynCom. *Bacillus megaterium* (9010) was not detected in the inoculum of SynCom 8A and *Frigobacterium sp* (9029) was not detected in the inoculum of SynCom 8B.

C) Transmission rate to seedlings (%) of each strain compared to its relative abundance in the inoculum (%).

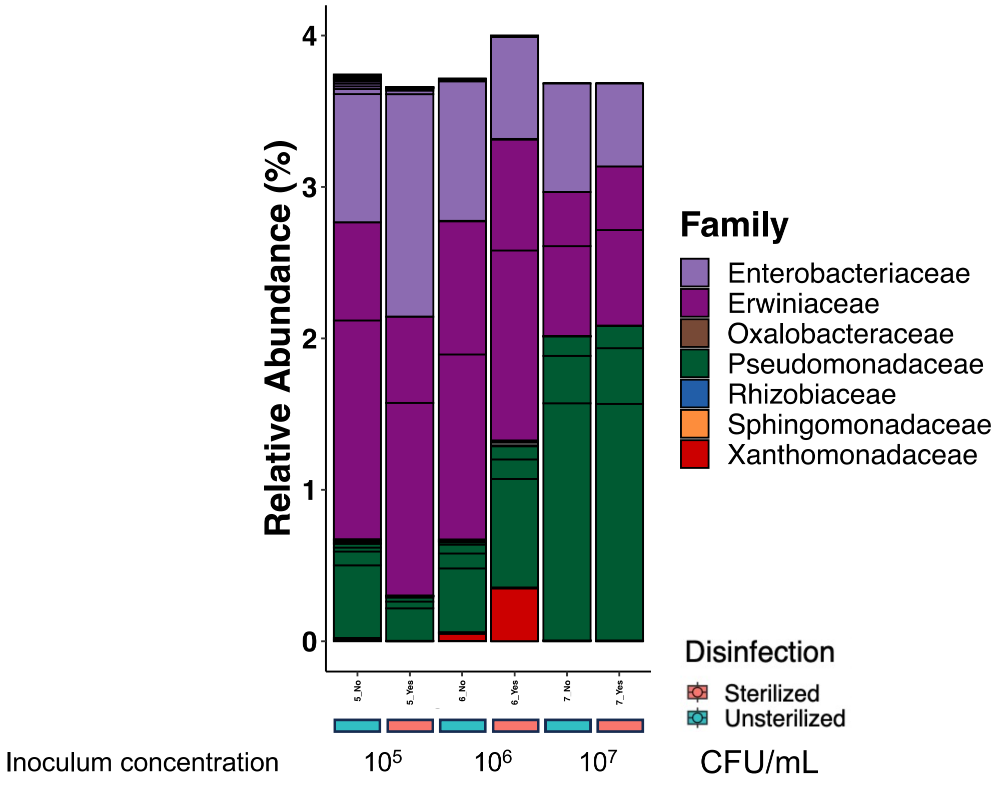

Figure S10:

Average taxonomic profiles of seeds from experiment 1 that do not belong to the SynCom14. For this analysis, all ASVs from SynCom14 were detected and removed to analyse only the native seed microbiota.
